# COVID-19 and the gendered impacts on adolescent wellbeing: Evidence from a cross-sectional study of locally adapted measures in Ethiopia, Jordan, and Palestine

**DOI:** 10.1016/j.eclinm.2022.101586

**Published:** 2022-08-03

**Authors:** Erin Oakley, Shoroq Abuhamad, Jennifer Seager, Benjamin Avuwadah, Joan Hamory, Nicola Jones, Agnieszka Małachowska, Workneh Yadete, Bassam Abu Hamad, Sarah Baird

**Affiliations:** a Department of Global Health, Milken Institute School of Public Health, George Washington University, United States; b Al-Quds University, Gaza, State of Palestine; c Department of Economics, University of Oklahoma, United States; d Gender and Adolescence: Global Evidence (GAGE), and ODI, London, UK; e Gender and Adolescence: Global Evidence (GAGE), UK; f Gender and Adolescence: Global Evidence (GAGE) and Quest Consulting, UK

**Keywords:** Adolescence, COVID-19, Gender, Wellbeing, Mixed methods

## Abstract

**Background:**

The COVID-19 pandemic and associated policy responses have interrupted services, increased financial stress, and driven social isolation, with acute impacts for adolescents. This study explores relationships between gender, COVID-19 vulnerability, social protection, and adolescent wellbeing in three diverse contexts: Ethiopia, Jordan, and Palestine.

**Methods:**

This study presents findings from a quantitative phone survey with adolescents in Ethiopia, Jordan, and Palestine (*n* = 5752) on household-level vulnerability to COVID-19-related shocks, household-level social protection (cash transfers or food aid), and locally adapted outcome measures designed to capture the gendered impacts of COVID-19 (collected between November 22, 2020 and February 25, 2021). We examine the relationship between each outcome and household-level COVID-19 vulnerability and social protection (and their interaction) using multivariate regressions, adjusting for adolescent and household characteristics.

**Findings:**

For all adolescents, increased vulnerability to COVID-19-related shocks is associated with worse outcomes for resilient coping and time spent on domestic tasks and care work. Across samples, girls spent over two additional hours on domestic and care work compared to boys. Girls in more vulnerable households experienced greater gendered constraints on behaviour. We find no association between receipt of social protection and adolescent wellbeing, and find that it only moderates the effect of COVID-19 vulnerability for less vulnerable households. Disability status, being out of school, and experiencing child marriage are also associated with adverse outcomes.

**Interpretation:**

Our study highlights that the pandemic has exacerbated underlying gender inequalities across adolescents in three very different settings, and that existing social safety nets are not adequate to fully address these impacts, particularly for the most vulnerable.

**Funding:**

This work was supported by UK aid through a grant from the Foreign, Commonwealth & Development Office to the Gender and Adolescence: Global Evidence (GAGE) longitudinal research study; the EMERGE project (Bill & Melinda Gates Foundation grants: OPP1163682 and INV018007; PI Anita Raj) and the World Health Organization (WHO) Regional Office for the Eastern Mediterranean.

**Research in context**

**Evidence before this study**

Research on adolescent wellbeing in low- and middle-income countries (LMICs) during the COVID-19 pandemic suggests that adolescents are at risk for adverse mental health, educational, and social outcomes. Through a rapid review of the literature surrounding adolescent time-use during the pandemic on PubMed, we identified few studies that examined adolescent time use in relation to domestic work and household responsibilities and wellbeing; overall, these studies find that many adolescents have increased the time spent on domestic or care work at home, and that large amounts of time spent on these tasks during the pandemic are associated with negative mental health outcomes.

**Added value of this study**

This study examines measures of adolescent wellbeing, including resilient coping, gendered constraints on behaviour, and time use during the COVID-19 pandemic in three LMICs: Ethiopia, Jordan, and Palestine. We used locally adapted survey measures to explore the impacts of the pandemic, finding significantly lower resilient coping and significantly higher time spent on household responsibilities for adolescents, as well as significantly higher level of gender-related restrictions on behavior for adolescent girls, from households considered more vulnerable to COVID-19. Overall, we found that receipt of social protection support was not associated with improved outcomes and did not mitigate the effects of COVID-19 vulnerability, except for adolescents from the least vulnerable households.

**Implications of all the available evidence**

Our results suggest that social safety nets in Ethiopia, Jordan, and Palestine have largely not been protective for adolescent wellbeing nor been able to mitigate the impact of pandemic shocks for the most vulnerable adolescents. Consistent with other studies of the gendered impacts of COVID-19 on time use, we find that adolescent girls experienced demands on their time and behavior in the home not seen among adolescent boys, particularly in households with the highest underlying COVID-19 vulnerability. Additional social protection programming, in the form of both greater economic support as well as age- and gender-responsive initiatives to address social disadvantages exacerbated by the pandemic, may therefore be necessary to foster recovery from pandemic shocks for vulnerable adolescents.

Alt-text: Unlabelled box

## Introduction

The COVID-19 pandemic and associated policy responses have interrupted services, increased financial stress, and driven social isolation around the world. For adolescents, these disruptions have been acute, resulting in multidimensional effects on wellbeing. Emerging research suggests that the pandemic has taken a toll on adolescent mental health and resilience in high-income countries (HICs) and low- and middle-income countries (LMICs) alike^1^^,^^2^ and that underlying inequities, such as poverty, are risk factors for worse outcomes.^3^ Moreover, studies suggest that girls may be at greater risk for adverse outcomes in terms of mental health^4^^,^^5^ and resilience.^6^ Studies of adolescent time use during the pandemic in LMICs also highlight how economically disadvantaged adolescents are particularly vulnerable to early entry into the labour force and/or high levels of household responsibility (such as child care).^2^^,^^7^^,^^8^ Gendered impacts on time use compound the mental health impacts of the pandemic; for women and girls, increases in (unpaid) domestic responsibilities such as child and elder care and household chores^9^^,^^10^ put them at greater risk of mental disorders, such as anxiety.^2^ Meanwhile, increased pressure to support the household through paid work may have negative impacts on boys’ wellbeing.^8^

Most studies on adolescent time use during the pandemic have focused on screen time and sedentary behaviour^11^^,^^12^ or on time spent on education during school closures, finding major decreases in time spent learning both in HICs^13^^,^^14^ and LMICs.^15^ There is sparse evidence from LMICs on the impact of the pandemic on girls’ time use for domestic chores and household responsibilities^3^; however, prior to the pandemic, the pattern of adolescent girls in LMICs spending significantly more time on household chores and care work than boys has been well-documented.^16^ This pattern may have persisted and worsened during the pandemic; for example, a study in Ecuador found that adolescent girls reported spending significantly more time on household tasks and care work than boys during school closures.^7^

This study examines multiple measures of adolescent wellbeing – each of which is adapted to the unique country context and assessed for validity and reliability – during the COVID-19 pandemic. We aim to understand the relationships betweengender, household vulnerability to COVID-19-related shocks, receipt of social protection, and adolescent wellbeing in three diverse LMIC contexts: Ethiopia, Jordan, and Palestine. Our measures of adolescent wellbeing focus on resilient coping, time use for domestic responsibilities, and gendered impacts on behavior in the home during the pandemic. As a component of the longitudinal Gender and Adolescence: Global Evidence (GAGE) study,^17^ this analysis builds on ongoing research assessing risk and protective factors for adolescent wellbeing during the pandemic.^18^, ^19^, ^20^, ^21^ Using data from 5,752 adolescents, we first developed locally adapted measures of adolescent wellbeing, following best practices for the development of valid and reliable measures.^22^^,^^23^ We used multivariate regression methods to understand the relationship between household-level COVID-19 vulnerability and our adolescent wellbeing measures, using an adapted measure of COVID-19 vulnerability across four domains: socioeconomic, demographic, epidemiological, and housing and hygiene. We then examined whether receipt of social protection (cash transfers or food aid) during the pandemic was associated with adolescent wellbeing and whether it mitigated adverse outcomes for adolescents from the households most vulnerable to COVID-19. Given that more vulnerable populations have fared worse under COVID-19, we hypothesise that adolescents from households with the highest levels of COVID-19 vulnerability will suffer the worst outcomes for wellbeing. Furthermore, we hypothesise that access to social safety nets will be associated with improved adolescent wellbeing and will act as a moderator of the relationship between COVID-19 vulnerability and adolescent wellbeing. Ultimately, understanding the role of vulnerability and access to social protection in supporting adolescent wellbeing during times of crisis can help inform gender- and age-responsive policies to tackle future crises.

This study examines the experiences of adolescents one year into the COVID-19 pandemic in three very different contexts: Ethiopia, Jordan, and Palestine. Even before considering the impact of the COVID-19 pandemic and mitigation strategies, there are vast differences in the lifestyles of adolescents living in different settings in each of these three contexts. Moreover, these countries had varied responses to the COVID-19 pandemic.

In Ethiopia, we consider adolescents from urban centres (Dire Dawa and Debre Tabor) and rural areas (South Gondar and East Hararghe). Nationally, Ethiopia has seen significant improvement over the last two decades in reducing poverty, increasing primary school completion, and reducing child marriage among girls under age 15.^24^ Even so, these improvements have not been uniform across regions. Disparities in secondary school enrollment persist among girls compared to boys even in urban areas; in rural areas, such as East Hararghe, adolescent girls are at higher risk for being kept home from school, whether for safety reasons, to work in the household, to marry, or because of gendered beliefs around the role of girls in mid- to late-adolescence.^24^ Globally, Ethiopia is one of the top three countries for the amount of time children and adolescent girls spend on work in the household.^16^ When domestic chores (such as collecting water and firewood, cooking, and cleaning) are considered alongside paid work and agricultural work, adolescent girls, and particularly those in poor and rural households, are much more likely than boys to spend a “harmful” amount of time working each day (defined as 4 hours per day or more), despite adolescent boys being more likely to be in the labor force.^25^

With the onset of the pandemic in March 2020, the Government of Ethiopia took several steps to prevent the spread of the virus, including communication campaigns to promote good hygiene and social distancing, restrictions on public gatherings, and school closures.^26^ Remote school via TV, radio, and social media was offered, but participation was very low outside of urban areas due to lack of access to technology.^27^ Schools in Ethiopia reopened in late 2020; however, the months-long interruption in schooling, as well as economic impacts of the pandemic on household budgets, meant that some adolescents did not return.^27^

In Jordan – an upper-middle-income country – some of the most vulnerable subpopulations in the country are refugees. As of January 2022, Jordan hosted 760,000 registered refugees, including over 670,000 Syrian refugees, as well as others from Palestine, Iraq, and other countries in the region.^28^ Most refugees live in host communities alongside the Jordanian population; however, nearly one fifth of Syrian refugees still reside in formal refugee camps, such as Azraq and Zaatari.^28^ A subgroup of communities—mainly Syrian refugees—live in informal tented settlements in rural areas of the country, often with less access to essential services, such as water, sanitation, healthcare, and education.^29^ Prior to the pandemic, GAGE research found that adolescent girls were more likely than boys to be enrolled in school; however, girls, and particularly Syrian refugee girls, are at greater risk of being married as children, which nearly always ends their education.^30^ Traditional gender norms limit future employment prospects for women and also leave adolescent girls and young women with a larger burden of unpaid domestic and care work in the home.^30^

At the onset of the COVID-19 pandemic in March 2020, the Government of Jordan (GOJ) quickly implemented strict measures to prevent the spread of the virus, including school closures, restrictions on travel and mobility, road closures, and curfews. Both case counts and mortality remained low through summer 2020, with just 12 deaths reported by August 2020; even so, the pandemic took a toll on the nation's economy. When restrictions were eased in late summer 2020, including the reopening of schools in September 2020, COVID-19 cases sharply increased. This lead the GOJ to reinstate various public health measures and close schools for most students through the 2020-2021 school year.^31^ During school closures, the Ministry of Education (MOE) offered remote schooling online and by TV, although some students struggled to continue learning due to lack of access to internet, particularly girls, who were less likely to have access to a personal mobile device.^27^

In the State of Palestine, we examine adolescent wellbeing during the COVID-19 pandemic in both Gaza and the West Bank. Prior to the pandemic, adolescents living in Palestine already faced the impacts of protracted conflict with Israel, economic isolation, and resource constraints, leading to high food insecurity and unemployment in the region.^32^^,^^33^ Conditions are particularly challenging in Gaza, where the international blockade on the region has severely restricted the mobility of people and resources for more than a decade.^34^ Nearly 4 in 10 residents of Palestine are refugees—more than 2.3 million people—with Gaza hosting a greater share of refugees than the West Bank.^33^ Although adolescent girls in Palestine tend to persist in school longer than their male peers, girls also face gender-based obstacles to their wellbeing, including patriarchal gender norms, restrictions on mobility and socialization from their families, a greater share of domestic responsibilities in the home compared to boys, and, in some cases, pressure to marry as children.^34^

Following the first cases of COVID-19 identified in the West Bank in early March 2020, a state of emergency was declared in both Gaza and the West Bank. Measures to stop the spread of the virus included closures of schools and religious institutions, as well as strict curfews and closure of primary healthcare centers to redirect resources toward treatment of COVID-19. In general, public health measures have been stricter in Gaza than in the West Bank; coupled with the isolating effects of the blockade and controlled entry to the region, the virus did not reach levels of community transmission in Gaza until August 2020.^35^ At the time of our survey (late 2020 and early 2021), Gaza was experiencing the highest number of COVID-19 cases to date, reaching the peak of its first wave in December 2020.^35^

## Methods

### Study design

This study presents findings from a cross-sectional survey conducted over the phone during the COVID-19 pandemic in late 2020 and early 2021, in Ethiopia, Jordan, and Palestine.

### Data source

Data was collected between November 2020 and February 2021 using a quantitative phone survey with 5,752 adolescents and youth aged 11–22 in Ethiopia (n = 2,301), Jordan (n = 2,534), and Palestine (n = 917).^1^ Research ethics approvals were obtained through the Overseas Development Institute's Research Ethics Committee (02438), and the George Washington University Committee on Human Research Institutional Review Board (071721). In Jordan, for research participants in refugee camps, we applied for and were granted permission from UNHCR's National Protection Working Group. For research participants in host communities, approval was granted by Jordan's Ministry of Interior, the Department of Statistics, and the Ministry of Education (19/29/92789/224879). In Ethiopia, approval was obtained from the Ethiopian Development Research Institute (EDRI/DP/00689/10); the Addis Ababa University College of Health Sciences Institutional Review Board (113/17/Ext); and the Amhara and Oromia regional health bureau ethics committees. In Palestine, permission was given from Gaza's Helsinki Committee (PHRC/HC/134/16). For all participants who were minors at the time of data collection, we collected informed consent from parents or guardians and informed assent from the adolescents themselves; for all participants who are emancipated (living independently) or who are over age 18, we collected informed consent from the respondent. Adolescents in Ethiopia and Jordan are part of the GAGE programme, a longitudinal study of adolescents in LMICs, and additional details on these samples are available in Jones et al. (2018).^36^ In Ethiopia, data was collected from a random sample of adolescents from selected rural zones (South Gondar and East Hararghe) and urban centres (Debre Tabor and Dire Dawa). In Jordan, the sample comprises Syrian refugee and Palestinian refugee adolescents living in refugee camps, host communities, or informal tented settlements, as well as vulnerable Jordanians and adolescents of other nationalities. The sample in Palestine is cross-sectional, and the sample frame was primarily provided by the Palestinian Central Bureau of Statistics based on the Multiple Indicator Cluster Survey (MICS) 2019/2020.^37^ Across all samples, we also conducted additional snowball sampling to recruit particularly vulnerable adolescents to the study, including adolescents with disabilities, girls who experienced child marriage, and out-of-school adolescents.

In Jordan, adolescents were initially enrolled in the GAGE study in late 2018 and early 2019. The sample used in the present analysis consists of 62% of the original sample, with lower participation by Jordanian adolescents than adolescents of other nationalities (Syrian and Palestinian refugees), but no other significant differences between the baseline and telephone samples. In Ethiopia, GAGE adolescents were initially enrolled in 2017-2018, and a large-scale follow-up survey was conducted in 2019 through early 2020. The telephone survey used in the present analysis includes 37% of the baseline sample, with higher representation of adolescents from urban areas, those enrolled in school, unmarried adolescents, and those from households with more assets – probably due to greater connectivity and mobile phone availability among these households. In Palestine, we employ a cross-sectional sample with enrolment in the study in late 2020. In Jordan and Palestine, phone access is close to universal (national estimates of household mobile phone ownership are 97.6% in Jordan^38^ and 97.3% in Palestine^39^); in Ethiopia, it is substantially lower (67.8% in our sample). Ultimately, the adolescents included in this study should be considered a convenience sample of vulnerable adolescents with phone access in these settings.

The survey instrument collected information on education, health and nutrition, mental health, mobility and social opportunities, paid work, and community impacts of COVID-19, as well as household-level details on resources and income, health and food security, and pandemic impacts (see the GAGE website for the full survey instruments^40^^,^^41^).

### Outcome measure development

Prior to implementing the phone survey, we adapted a set of measures to explore the varied impacts of the COVID-19 pandemic on adolescent wellbeing. We began with modules from the Evidence-Based Measures of Empowerment for Research on Gender Equality (EMERGE) COVID-19 and Gender Survey Questions compiled and adapted by the Center on Gender Equity and Health^42^ related to Women and Girls’ Agency^43^ and Domestic Work Distribution, Time Use and Unpaid Labor.^44^ These measures are: (1) the ***Brief Resilient Coping Scale (BRCS)***, a 5-item scale that includes the original four items developed by Sinclair and Wallston^45^ that measure “tendencies to cope with stress in a highly adaptive manner,” and one additional COVID-related item added by the EMERGE project; (2) the ***Gendered Constraints on women and girls’ Behaviors Scale (GCBS)****,* a series of five questions asked to women and girls on how they have had to change their behaviour at home “due to the increased presence of men in the household following the COVID-19 pandemic and the social containment efforts to manage the spread of the virus” developed by EMERGE^44^; and (3) the ***Domestic Work Distribution (DWD)*** module, which asks respondents about six domestic and care work activities and how time spent on these activities has changed following the pandemic and was adapted by the EMERGE team from the UN Women Rapid Assessment Survey on the socioeconomic consequences of COVID-19 on women's and men's economic empowerment.^46^

### Adapting the measures

We adapted these measures to our population (adolescents) and settings (Ethiopia, Jordan, and Palestine) using formative qualitative and cognitive interviews, drawing on the EMERGE guidelines for creating and adapting valid social and behavioural measures on gender equality and empowerment.^22^^,^^23^ We began the adaptation process through formative qualitative in-depth interviews with adolescents and key informants in each setting (139 adolescents and 43 key informants in total), focusing on key topics related to the EMERGE survey modules, including adolescent coping strategies, intra-household gender and age divisions of labour, and intra-household relations during COVID-19-related shutdowns. (See Appendix A, Qualitative Interviews for full details on these interviews and analysis methods.)

We then adapted the BRCS by adding four items relevant to COVID-19. We added two items relevant to adolescents to the GCBS and adapted the language on the original items to be more inclusive of possible constraints. The DWD module underwent more substantial adaptation: the team added four time categories, and it was modified to capture both the time spent by adolescents on each category and perceptions of how time use has changed since the pandemic.

Survey teams in each setting then administered a series of cognitive interviews with these adapted measures (with 46 adolescents in Ethiopia, 62 in Jordan, and 20 in Palestine), asking adolescents each question, as well as their understanding of the question and its answer options. We incorporated feedback from this process into the adapted measures before launching a pilot version of the full survey. (See Appendix A, Cognitive Interviews for the final version of the adapted EMERGE module measures used in each setting, with additional details on the qualitative research, cognitive interview procedures and samples.)

### Psychometric methods

Where applicable, we use Cronbach's alpha to assess internal reliability of the locally-adapted measures.^23^^,^^47^ Further, we conducted a follow-up re-test with a small sample of adolescents in Ethiopia and Jordan in the weeks following the initial administration of the survey. Using this follow-up sample, we conducted several tests of external validity and stability: for all scored modules, we calculated an intraclass correlation coefficient (ICC) using a two-way mixed-methods model to assess absolute agreement in survey measures over time,^48^ as well as a test-retest correlation (Pearson's correlation,^23^), and for categorical modules we use Cohen's kappa.^49^^,^^50^

### Outcomes

Six key outcomes emerge from the locally adapted survey modules. First, we calculated each adolescent's total score on the original 4-item BRCS (BRCS-O, calculated on a scale of 0–16). Second, we calculated each adolescent's total score on the five new items added to the BRCS using the same values for answer options as the original scale (creating a scale of 0–20, the BRCS-COVID-19).^2^ Next, we constructed three variables from the DWD module reflecting the amount of time spent in the past 24 hours on domestic responsibilities, including: (1) sum of hours spent on domestic tasks and care work, including cooking, cleaning, elder or child care, caring for sick relatives, and helping children or siblings with school work; (2) sum of hours spent on work outside the household and income-generating tasks, including agricultural work, working in a family business, or paid work outside the home; and (3) a joint measure of “all domestic responsibilities”, which is the sum of the two previous categories.^3^ Finally, we created an index of the number of constraints reported by each female adolescent in the GCBS, with a total possible score of 7.^4^ All outcomes are standardised to the mean and standard deviation within each country in the regression analysis.

### COVID-19 vulnerability and social protection

We focus on two main household-level independent variables that may influence adolescent wellbeing outcomes during the pandemic. The first is an index of pandemic vulnerability factors (measured on a scale of 0–9), where each of the following characteristics increases the household's vulnerability score by 1: household head has less than secondary education; household reports that either cost or distance is a barrier to accessing health care; household lives in fewer than three rooms; household does not have its own toilet; adolescent does not have access to water or soap for handwashing most of the time; any household member has a chronic health condition that increases COVID-19 vulnerability; any household member is aged 60 years or older; any household member is currently or recently pregnant; and household lacks any of a set of context-specific selected assets.^5^ These measures capture COVID-19 vulnerability across four domains: socioeconomic, demographic, epidemiological, and housing and hygiene. To account for differences in vulnerability factors by location, we standardised these indices within each country in the regression analysis.

The second independent variable is a measure of social protection during the pandemic from a large-scale safety net programme. In Ethiopia, we examine current aid received through the government's Productive Safety Net Programme (PSNP), designed to improve household food security.^51^ In Jordan, we examine current aid provided through any of four possible programmes: (1) World Food Programme (WFP) food aid vouchers distributed to Syrian and other refugee households^52^; (2) cash transfers provided by the United Nations High Commissioner for Refugees (UNHCR) to Syrian and other refugee households^53^; (3) food aid and cards provided by the United Nations Relief and Works Agency for Palestine Refugees in the Near East (UNRWA) to vulnerable Palestinian refugees living in Jordan^54^; and (4) Hajati cash transfers to vulnerable Jordanian and non-camp refugee households with school-age children.^55^ In Palestine, we examine current support through UNRWA food aid and food aid e-cards, provided to vulnerable refugee families in Gaza and the West Bank respectively,^54^ and WFP vouchers for food-insecure, non-refugee households.^32^

### Covariates

Individual and household characteristics considered in this analysis include the adolescent's gender, age, school enrolment status (in March 2020), household wealth (defined as being above or below a country-specific median asset index score, constructed using methods of Filmer and Pritchett^56^), marital status (ever- or never-married), disability status (with or without a functional disability [seeing, hearing, walking, remembering, self-care]), and country-specific location and nationality indicators.^6^ We adjust for these covariates as confounders in the relationship between COVID-19 vulnerability and social protection and adolescent wellbeing, as well as to identify additional risk factors for adverse outcomes.

### Statistical analysis

Descriptive statistics (means and standard deviation) for each outcome were generated for the pooled sample and disaggregated by gender and country. Bivariate differences by gender within country were tested for using two-sided t-tests. Multivariate linear regression was used to estimate the association between our outcomes of interest and COVID-19 vulnerability and receipt of social protection. We also tested for the interaction between COVID-19 vulnerability and receipt of social protection to assess whether access to social protection moderates the role of COVID-19 vulnerability. All models adjust for the previously mentioned covariates to control for potential confounders. Standard errors are clustered at the *subkebele*^7^ level in Ethiopia to account for sampling design, and at the individual level in Jordan and Palestine, where clustered sampling was not employed. All statistical analyses were conducted in Stata version 16.1.

### Role of the funding source

The funders of the study had no role in study design, data collection, data analysis, data interpretation, or writing of the report. All authors had full access to the data in the study and had final responsibility for the decision to submit for publication.

## Results

### Psychometric results

#### Internal reliability

Within our full sample of 5,752 adolescents, the two adapted scale-based measures (the BRCS and GCBS) showed moderate-to-acceptable internal reliability across samples^47^ and attained similar internal reliability estimates as the original BRCS measure.^45^ Cronbach's alpha for the BRCS-O was 0·68 across samples and 0·64 for the BRCS-COVID-19. Considered together, all nine items from the coping module attained an alpha of 0·80 across settings, indicating acceptable internal reliability. Cronbach's alpha for the GCBS was 0·81 across samples (0·82 in Ethiopia, 0·75 in Jordan, and 0·79 in Palestine), also suggesting acceptable internal reliability.

### External reliability and measurement stability

We re-administered all three adapted modules to a subsample of adolescents at the conclusion of the main data collection period in Ethiopia (n = 180, including n = 44 retests of the BRCS-O and BRCS-COVID-19) and Jordan (n = 64) to examine external reliability. Retests occurred 2 to 7 weeks after the respondent completed the original survey in Jordan, and 1 to 10 weeks after in Ethiopia. For scored modules (BRCS-O, BRCS-COVID-19, and GCBS), we calculated an ICC using a two-way mixed-methods model to assess absolute agreement in survey measures over time,^48^ as well as a test-retest correlation (Pearson's correlation,^23^). For the DWD module, we examined external reliability and stability of categorical questions (i.e., those focused on perceived change in time use compared to before the pandemic) using Cohen's kappa.^49^^,^^50^ (See Appendix A, Psychometrics for additional details on test-retest methods and country-specific findings.)

Overall, we find low test-retest agreement and correlation for the BRCS-O, the BRCS-COVID-19, and the 9-item scale overall; ICC estimates for the 9-item scale overall were 0·21 in Jordan (95% CI: -0·05, 0·43) and 0·10 in Ethiopia (95% CI: -0·19, 0·38), with lower agreement on the BRCS-COVID-19 than the original 4-item scale. We suspect that several factors play a role in these results; survey respondents were experiencing pandemic-related shocks, potentially causing greater real changes in reported measures of resilient coping over time than found in previous studies of the BRCS.^45^ We also acknowledge that the variable amount of time between test and retest for each adolescent is not ideal for examining external reliability.

For the GCBS, we find higher agreement in responses over time among girls in Jordan (ICC 0·46, 95% CI: 0·15, 0·69), but no agreement over time in Ethiopia, which may be partly due to the low levels of gendered constraints on behaviour reported in the sample overall; the mean score on the 7-point scale in Ethiopia ranged from 0·87 in the initial administration to 1·39 in the retest for this subsample.

For the DWD module, kappa ranged from 0·208 to 0·465 in Jordan for all domestic responsibilities except for shopping (0·075) and working in a household business (−0·012), suggesting fair-to-moderate levels of stability over the survey period. In Ethiopia, we found fair-to-moderate stability for tasks including agricultural work (0·529), cooking (0·431), cleaning (0·296), working for a household business (0·289), and child care (0·251), with little or no stability for other tasks.

Ultimately, for our sample, BRCS and related coping questions should not be considered a stable measure of resilient coping over time, given the changes observed in this subsample in a relatively short period of time. The GCBS and DWD items showed slightly higher levels of external reliability; however, the current survey measures did not demonstrate adequate stability over the time period examined. This may be somewhat expected, as these outcomes (apart from the original 4-item BRCS) were created to measure gendered impact of the COVID-19 pandemic on day-to-day life—an experience that may be perceived differently over time. Ultimately, while these measures show good internal reliability, measurement stability is low, which is likely indicative of measure development of constructs in contexts of ongoing crisis.

### Descriptive statistics

Table 1 presents descriptive statistics for the analysis sample of 5,752 adolescents. The average age of adolescents is 15·4 and about half of the sample is female (52%), with variation by country. Across samples, the share of adolescents who have ever been married ranges from 6·7% in Jordan to 8·5% in Palestine, while the rate of adolescents self-identifying as having a disability ranged from 4·7% in Ethiopia to 17·1% in Palestine. In terms of COVID-19 vulnerability, the mean of the raw index was higher in Ethiopia than Jordan and Palestine (3·4 vs 2·9 and 2·3, respectively). Rates of household-level social protection vary widely by sample. Whereas in Ethiopia, just 11% of households reported currently benefiting from the PSNP, in Jordan 80% of households reported currently receiving some form of aid – probably driven in part by the large-scale social protections provided to Syrian refugees, who have limited opportunities for work and were more likely to live below the poverty line and rely on social assistance prior to the pandemic.^57^^,^^58^ In Palestine, 41% of households reported currently receiving aid.

**Table 1:** Demographics, COVID-19 vulnerability, and social protection by country.

	Ethiopia		Jordan		Palestine	
	Mean	SD	Mean	SD	Mean	SD
*Panel A: Demographics*						
Female	0.548	0.498	0.506	0.500	0.489	0.500
Age (11–22)^a^	15.299	2.524	15.492	2.583	15.297	2.146
Household is above median on asset index *	0.630	0.483	0.416	0.493	0.448	0.498
Adolescent has ever been married	0.081	0.273	0.067	0.250	0.085	0.279
Adolescent has a disability	0.047	0.212	0.140	0.348	0.171	0.377
Adolescent is enrolled in school	0.907	0.290	0.713	0.453	0.786	0.410
*Panel B: Country-specific details*						
Ethiopia: Dire Dawa (urban)	0.204	0.403	–	–	–	–
Ethiopia: Debre Tabor (urban)	0.269	0.443	–	–	–	–
Ethiopia: South Gondar (rural)	0.285	0.451	–	–	–	–
Ethiopia: East Hararghe (rural)	0.243	0.429	–	–	–	–
Jordan: Adolescent is Jordanian	–	–	0.123	0.329	–	–
Jordan: Adolescent is a Palestinian refugee living in Jordan	–	–	0.078	0.268	–	–
Jordan: Adolescent is a Syrian refugee living in a camp	–	–	0.230	0.421	–	–
Jordan: Adolescent is a Syrian refugee living in a host community	–	–	0.468	0.499	–	–
Jordan: Adolescent is a Syrian refugee living in informal tented settlement (ITS)	–	–	0.086	0.281	–	–
Jordan: Adolescent is other nationality living in Jordan	–	–	0.014	0.118	–	–
Palestine: Gaza	–	–	–	–	0.542	0.499
Palestine: West Bank	–	–	–	–	0.458	0.499
Palestine: Adolescent is a refugee	–	–	–	–	0.288	0.453
Palestine: Adolescent lives in a refugee camp	–	–	–	–	0.486	0.500
*Panel C: COVID-19 vulnerability*						
Index of COVID-19 vulnerability (0–9), higher is more vulnerable	3.425	1.513	2.898	1.367	2.301	1.562
Household lacks selected assets (setting-specific)^b^ *	0.126	0.332	0.066	0.248	0.273	0.446
Household head does NOT have secondary qualification or higher *	0.814	0.389	0.752	0.432	0.448	0.498
Cost or distance is barrier to health care *	0.456	0.498	0.660	0.474	0.565	0.496
Household has fewer than 3 rooms *	0.684	0.465	0.479	0.500	0.320	0.467
Household does not have own toilet *	0.572	0.495	0.156	0.363	0.108	0.311
Adolescent does not have access to water or soap most or all of the time	0.301	0.459	0.122	0.327	0.059	0.236
Any household member(s) has a chronic health condition	0.186	0.390	0.429	0.495	0.352	0.478
Any household member(s) is over 60 years old	0.203	0.402	0.131	0.337	0.099	0.299
Any household member(s) is pregnant or recently gave birth	0.081	0.273	0.103	0.304	0.077	0.267
*Panel D: Current receipt of social protection*						
Household receives social protection	0.113	0.316	0.798	0.402	0.414	0.493
Sample size	2,301		2,534		917	

*Notes:* This table presents means (and standard deviation) for demographic variables of interest collected during the COVID-19 phone survey (November 2020 to February 2021). Variables marked * were collected for the Jordan sample during baseline data collection (October 2018 to March 2019) and for Ethiopia during baseline (December 2017 to May 2018) and midline (December 2019 to May 2020) data collection. The Index of COVID-19 vulnerability is the sum of the subsequent indicators in Panel C.

a A small number of adolescents (*n*=10) in Ethiopia reported an out-of-range age for this survey (age 23–25). Aside from these outliers, 99.8% of the sample are aged 11–22.

b In Ethiopia, selected assets used for this index include “TV or radio, fridge, or mattress”. In Jordan, selected assets include “fridge or fan/AC”, and in Palestine “TV, fridge or fan/AC”.

Table 2 presents summary statistics for outcomes of interest. The average BRCS-O scores are slightly higher for boys (10·383 [SD 2·977]) than girls (10·183 [SD 3·020]), particularly in Jordan, where girls’ average score is 0·396 lower than that of boys (see Table 2). Likewise, scores on the BRCS-COVID-19 are similar by gender in Ethiopia and Palestine, while boys again score 0·358 points higher than girls in Jordan. The GCBS scores exhibit significant differences by country (Table 2). Girls in Ethiopia experience the fewest constraints on behaviour, with an average of less than 1 constraint reported (0·615 [SD 1·240]), while girls in Jordan report the highest number of constraints (2·181 [SD 1·781]).

**Table 2:** Outcome means (SD) by sample and gender – BRCS, GCBS, and time use (in hours).

	Ethiopia		Jordan		Palestine		Global		
	Boys	Girls	Boys	Girls	Boys	Girls	Boys	Girls	N^a^
BRCS-O (0–16)	10.683 (2.771)	10.639 (2.786)	10.223 (3.115)	9.827*** (3.166)	10.482 (2.788)	10.578 (2.760)	10.383 (2.977)	10.183** (3.020)	4,577
BRCS-COVID-19 (0–20)	12.753 (3.770)	12.532 (3.483)	12.960 (3.262)	12.602*** (3.237)	12.676 (3.002)	12.881 (2.926)	12.853 (3.336)	12.637** (3.249)	4,581
GCBS (0–7)	–	0.615 (1.240)	–	2.181 (1.781)	–	1.262 (1.561)	–	1.362 (1.696)	2,864
**Time per day spent on…**									
Domestic work and care tasks	2.559 (2.689)	4.442*** (2.609)	3.481 (3.343)	5.797*** (4.187)	1.381 (2.359)	3.816 (3.631) ***	2.772 (3.047)	4.922*** (3.594)	5,700
Agriculture and paid work	1.838 (2.549)	0.904*** (1.976)	1.719 (3.666)	0.254*** (1.499)	1.060 (3.193)	0.033 (0.455) ***	1.652 (3.215)	0.495*** (1.663)	5,734
Domestic or ag/paid work	4.397 (4.106)	5.346*** (3.314)	5.198 (4.887)	6.049*** (4.418)	2.441 (3.829)	3.849*** (3.662)	4.423 (4.536)	5.418*** (3.937)	5,700
**Time per day spent on… (among those enrolled in school in March 2020)**									
Domestic work and care tasks	2.579 (2.692)	4.343*** (2.518)	3.614 (3.263)	5.189*** (3.896)	1.466 (2.441)	3.253*** (3.173)	2.805 (2.988)	4.502*** (3.278)	4,581
Agriculture and paid work	1.785 (2.458)	0.803*** (1.722)	1.298 (3.150)	0.213*** (1.351)	0.432 (2.066)	0.041*** (0.502)	1.377 (2.739)	0.457*** (1.487)	4,604
Domestic or ag/paid work	4.364 (4.090)	5.146*** (3.131)	4.912 (4.530)	5.404** (4.124)	1.898 (3.245)	3.293*** (3.223)	4.182 (4.274)	4.960*** (3.630)	4,581
Studying/ learning in the past week	3.545 (2.791)	3.580 (2.440)	2.453 (2.363)	3.045*** (2.567)	2.326 (1.838)	3.136*** (2.223)	2.918 (2.554)	3.301*** (2.471)	4,587

*Notes:* This table presents mean and standard deviation of the BRCS-O, BRCS-COVID-19, GCBS, and mean time use in hours (with standard deviation) reported for the past 24 h on the DWD module by all adolescents and, in the final panel, by adolescents who were enrolled in formal schooling in March 2020. In the final panel, we also present an additional time use variable collected in the survey, which asked students the typical number of hours spent "studying or learning" during each weekday in the past 7 days (i.e., while schools were closed). Within each country and for the sample overall, we tested for significant differences in outcomes for boys and girls using two-sided t-tests. Asterisks indicate where responses were statistically significantly different for girls than for boys (*** *p*<.01, ** *p*<.05, * *p*<.10).

a The overall global sample size is *n*=5752. Sample sizes vary slightly by outcome because any questions where the adolescent refuses the question or responds "Don't know" is excluded from that outcome measure. In addition, the BRCS-O and BRCS-COVID-19 were randomly administered to a random subsample of approximately one-third of the total Ethiopian sample due to time constraints, resulting in a smaller global sample size. The sample sizes for Ethiopia for these outcomes are *n*=1384 and *n*=1385 respectively. Finally, the sample size for the outcome “Index of gendered constraints on behaviour” is restricted to female respondents only.

For time spent on domestic work and household responsibilities, patterns are strongly gendered (see Table 2). Across all adolescents, girls spent an average of 4·922 hours (SD 3·594) on domestic tasks and care work in the past 24 hours – nearly double the hours reported by boys (2·772 [SD 3·047]). In contrast, adolescent boys spent nearly three times as many hours as girls on agriculture, working for a household business, or other paid work (1·652 hours among boys, [SD 3·215]). However, even considering this additional time spent on agricultural and paid work tasks, girls spent almost 1 more hour per day (5·418 hours [SD 3·937]) than boys (4·423 hours [SD 4·536]) on all household responsibilities. Among those currently enrolled in school, both boys and girls reported spending more hours on household responsibilities than they spent on learning in a typical day.

### Multivariate regression analysis

Table 3 presents multivariate regression results for the pooled sample of all countries. COVID-19 vulnerability is associated with decreases in adolescent resilient coping and increases in time spent on domestic and care work. In Row 1, a one standard deviation (SD) increase in COVID-19 vulnerability (1·5 points) is associated with a decrease in the BRCS-O by 0·042 SD (CI −0·074, −0·011) and the BRCS-COVID-19 by 0·038 SD (CI −0·069, −0·007). In terms of time use, a one SD increase in COVID-19 vulnerability is associated with a 0·055 SD (CI 0·028, 0·082) increase in domestic and care work, equivalent to an increase of about 10 minutes per day, but is not associated with time spent on agriculture or paid work. In the second and third rows of Table 3, we explore whether receiving social protection is associated with resilient coping and adolescent time use, and protective against greater COVID-19 vulnerability. We find no association between receiving social protection and any of our outcomes of interest. Further, we find that adolescents in households that have greater COVID-19 vulnerability and receive social protection spend more time on domestic work and care tasks than adolescents receiving social protection in less vulnerable households (column 3). Looking deeper into the data, social protection reduces time spent on domestic work and care tasks for girls in households with below-average vulnerability, but has no such effect for girls in more vulnerable households (not shown).

**Table 3:** Multivariate regression results for the global sample – COVID-19 vulnerability, social protection, and interaction.

	(1)	(2)	(3)	(4)	(5)
	BRCS-O	BRCS-COVID-19	Domestic work	Ag/paid work	Domestic or ag/paid work
1. COVID-19 vulnerability	−0.042***	−0.038**	0.055***	0.018	0.057***
	(0.016)	(0.016)	(0.014)	(0.014)	(0.014)
2. Received social protection	0.016	0.043	−0.052	0.045	−0.012
	(0.054)	(0.052)	(0.044)	(0.038)	(0.041)
3. Vulnerable x social protection	0.049	0.029	0.090***	−0.022	0.067**
	(0.031)	(0.032)	(0.028)	(0.027)	(0.028)
Number of observations	4,577	4,581	5,700	5,734	5,700
Unstandardised outcome mean and SD					
Mean	10.281	12.742	3.890	1.050	4.940
SD	3.000	3.293	3.511	2.594	4.264

*Notes.* This table uses data for girls and boys across all countries (Ethiopia, Jordan, and Palestine). Each panel presents coefficient estimates from a separate specification and each cell presents coefficients from a separate estimation. Standard errors are given in parentheses below each coefficient estimate. Outcomes of interest are labelled at the top of each column, and independent variables of interest are labelled at the start of each row. All outcomes are standardised to the mean and standard deviation within country. In columns 1 and 2, the BRCS-O is the standardised sum of the adolescent's total score on the original 4-item BRCS and the BRCS-COVID-19 is the standardised sum of the adolescent's total score on the 5 COVID-19-related coping items. For Ethiopia, the BRCS measures were administered to a random subgroup of one-third of the total sample, resulting in a smaller number of respondents for these outcomes (columns 1 and 2). In column 3, the outcome is the standardised number of hours the adolescent reports spending on domestic tasks and care work. In column 4, the outcome is the standardised number of hours the adolescent reports spending on work outside the household and income-generating tasks, including time spent on agricultural work for the household, working in a family business, or engaging in paid work outside the home. The outcome in column 5 is the standardised sum of the total number of hours reported for tasks in columns 3 and 4. The mean and standard deviation of the unstandardised outcome is provided at the bottom of the table. Each regression includes controls for the adolescent's age, gender, wealth, marital status, disability status, school enrolment status (in March 2020), and a set of location and ethnicity indicators for each country (see Table 1, Panel B). Standard errors are clustered at the *subkebele* level in Ethiopia to account for sampling design, and at the individual level in Jordan and Palestine. *** p<.01, ** p<.05, * p<.10.

Appendix Table B1 presents covariate estimates for each outcome to explore other sources of variation in our outcomes of interest. Girls have lower BRCS-O scores than boys, on average, and gender and age are strongly predictive of time spent in domestic work (girls) and time spent in paid work (boys). We find that ever-married girls spend the most time on domestic work. Interestingly, ever-married adolescents have higher BRCS-O scores. Adolescents in wealthier households also have higher BRCS-COVID-19 scores and are less likely to engage in domestic work. Adolescents who were enrolled in school prior to the pandemic have higher BRCS-O and BRCS-COVID-19 scores on average and are less likely to be engaged in paid work.

In Table 4, we present regression results separately for girls (Panel A) and boys (Panel B). This allows us to identify gender differences in the relationship between COVID-19 vulnerability and receipt of social protection and adolescent coping and time use, as well as to explore their relationship with the GCBS (for girls). Table 4 shows that COVID-19 vulnerability and receipt of social protection have similar associations with the BRCS and DWD time use measures looking separately by gender as they do in the whole sample, with a couple of exceptions. For girls, a one SD increase in COVID-19 vulnerability is associated with a 0·041 SD (CI 0·017, 0·065) increase in time spent in agriculture or paid work (approximately 5 additional minutes per day on average). COVID-19 vulnerability is also strongly positively associated with the GCBS (0·121 SD (0·079, 0·164)); as with other outcomes, receipt of social protection does not appear to play a mitigating role for COVID-19 vulnerability on average.

**Table 4:** Multivariate regression results for the global sample by gender – COVID-19 vulnerability, social protection, and interaction.

	(1)	(2)	(3)	(4)	(5)	(6)
	BRCS-O	BRCS-COVID-19	Domestic Work	Ag/paid work	Domestic or ag/paid work	GCBS
*Panel A: Adolescent girls*						
1. COVID-19 vulnerability	−0.039*	−0.048**	0.055***	0.041***	0.071***	0.121***
	(0.023)	(0.023)	(0.020)	(0.012)	(0.018)	(0.022)
2. Received social protection	0.069	0.065	−0.020	0.019	0.002	0.012
	(0.077)	(0.074)	(0.064)	(0.038)	(0.056)	(0.065)
3. Vulnerable x social protection	0.044	0.003	0.127***	−0.019	0.099***	0.069*
	(0.044)	(0.044)	(0.040)	(0.024)	(0.036)	(0.041)
Number of observations	2,345	2,348	2,964	2,984	2,964	2,864
Unstandardised outcome mean and SD						
Mean	10.281	12.742	3.890	1.050	4.940	1.362
SD	3.000	3.293	3.511	2.594	4.264	1.696

	(1)	(2)	(3)	(4)	(5)	(6)
	BRCS-O	BRCS-COVID-19	Domestic Work	Ag/paid work	Domestic or Ag/paid work	GCBS
*Panel B: Adolescent boys*						
1. COVID-19 vulnerability	−0.048*	−0.029**	0.056***	−0.006***	0.043***	–
	(0.023)	(0.023)	(0.019)	(0.025)	(0.022)	–
2. Received social protection	−0.057	0.007	−0.074	0.056	−0.029	–
	(0.072)	(0.076)	(0.061)	(0.070)	(0.064)	–
3. Vulnerable x social protection	0.058	0.067	0.050***	−0.012	0.041***	–
	(0.046)	(0.048)	(0.037)	(0.047)	(0.042)	–
Number of observations	2,232	2,233	2,736	2,750	2,736	–
Unstandardised outcome mean and SD						
Mean	10.383	12.853	2.772	1.652	4.423	–
SD	2.977	3.336	3.047	3.215	4.536	–

*Notes.* This table uses data for girls in Panel A and boys in Panel B across all countries (Ethiopia, Jordan, and Palestine). Each sub-panel presents coefficient estimates from a separate specification and each cell presents coefficients from a separate estimation. Standard errors are given in parentheses below each coefficient estimate. Outcomes of interest are labelled at the top of each column, and independent variables of interest are labelled at the start of each row. All outcomes are standardised to the mean and standard deviation within country. In columns 1 and 2, the BRCS-O is the standardised sum of the adolescent's total score on the original 4-item BRCS and the BRCS-COVID-19 is the standardised sum of the adolescent's total score on the 5 COVID-19-related coping items. For Ethiopia, the BRCS measures were administered to a random subgroup of one-third of the total sample, resulting in a smaller number of respondents for these outcomes (columns 1 and 2). In column 3, the outcome is the standardised number of hours the adolescent reports spending on domestic tasks and care work. In column 4, the outcome is the standardised number of hours the adolescent reports spending on work outside the household and income-generating tasks. The outcome in column 5 is the standardised sum of the total number of hours reported for tasks in columns 3 and 4. In column 6, the outcome is the standardised measure of the GCBS, the total number of constraints reported by each female adolescent. The mean and standard deviation of the unstandardised outcome is provided at the bottom of the table. Each regression includes controls for the adolescent's age, gender, wealth, marital status, disability status, school enrolment status (March 2020), and a set of location and ethnicity indicators for each country (see Table 1, Panel B). Standard errors are clustered at the *subkebele* level in Ethiopia to account for sampling design, and at the individual level in Jordan and Palestine. *** *p*<.01, ** *p*<.05, * *p*<.10.

For boys and girls, marital status emerges as a significant predictor of resilient coping and time use; ever-married boys spent an average of 2 additional hours (0·698 SD; CI 0·073, 1·323) on work tasks compared to never-married boys, while ever-married girls spent an average of 1·5 additional hours (0·425 SD; CI 0·278, 0·571) on domestic and care tasks compared to never-married girls, and both ever-married boys and girls have significantly higher resilient coping scores on at least one measure (see Appendix B, Table B2 and B3). Disability status also appears to have some differential effects by gender; among boys, disability status is associated with a significant reduction in BRCS-O scores (−0·147 SD; CI −0·278, −0·017). Among girls, disability status does not appear to have such a significant effect on measures of resilient coping; however, disability status is associated with a significant increase in gendered restrictions on behaviours in the home (0·147 SD; CI 0·025, 0·269).

Notably, several country-specific location and nationality indicators have a significant impact on adolescent outcomes. To explore these dynamics further, we repeated all estimations separately within each country sample (see Appendix B, Tables B4-B12).

In Ethiopia, receipt of social protection continues to have little impact on adolescent BRCS and DWD outcomes or on GCBS outcomes for girls. Zone of residence is associated with significant differences in both BRCS and GCBS scores: living in East Hararghe (a rural area) or Dire Dawa (an urban centre) was associated with a small increase in BRCS-O and BRCS-COVID-19 scores, while girls in East Hararghe and Dire Dawa had higher scores on the GCBS than girls in South Gondar (a rural area) and Debre Tabor (another urban centre). In Jordan, receipt of social protection is associated with increased BRCS-O scores for adolescent girls (0·343 SD; CI 0·090, 0·535), but not for boys, and is associated with decreased time spent on domestic work and care tasks for boys (0·227 SD; CI −0·482, 0·028), but not girls. Furthermore, compared to their Jordanian and Palestinian peers, Syrian refugee adolescents report higher BRCS-COVID-19 scores across host communities, camps, and ITSs, with Syrian girls in particular experiencing a protective effect for BRCS-COVID-19 scores from living in a refugee camp.

In Palestine, we observed significant differences in resilient coping and time use for adolescents in Gaza compared to those in the West Bank, with the former displaying lower BRCS-O scores. Gazan boys and girls also reported significantly more hours spent on domestic work, and Gazan girls had higher GCBS scores, indicating higher average levels of gendered constraints on behaviour. Further, COVID-19 vulnerability was strongly associated with increased GCBS for girls and decreased BRCS scores, both overall and for boys.

## Discussion

We find that COVID-19 vulnerability has consistent and negative impacts on several dimensions of adolescent wellbeing, and that social safety nets designed to ease the economic burden of crises, such as the COVID-19 pandemic, are not sufficient to mitigate these negative impacts for the most vulnerable households. Specifically, we find that adolescents in households that are more vulnerable to the impacts of COVID-19—measured in terms of socioeconomic, demographic, epidemiological, and housing and hygiene vulnerability—exhibit lower resilient coping, and spend more time on domestic and care work (boys and girls alike), while girls in more vulnerable households also spend more time on agriculture and paid work and face more gendered constraints on behaviour. Receipt of social protection, on the other hand, is not associated with adolescent wellbeing overall, and appears to be protective only for households with below-average vulnerability. This suggests that existing social protection programmes may not be sufficient to promote the wellbeing of the most vulnerable adolescents during and after the pandemic.

It is worth noting that most of the large-scale social protection programmes reviewed in this study provide household-level support. Only Hajati cash transfer aid in Jordan is targeted for individual children and adolescents specifically, as this program is intended as an unconditional cash transfer to support school enrollment.^55^ Whether adolescent-specific aid has a differential impact on adolescent wellbeing during a time of crisis is an interesting area for future research.

Our findings expand several strands of research on the multidimensional impacts of the COVID-19 pandemic on adult men and women to the adolescent population. We provide new evidence that the additional responsibilities in the home placed on women during the pandemic – well-documented as a major pandemic stressor in all contexts^9^^,^^10^^,^^46^ – also applies to adolescents in LMICs. We observe that adolescent girls in Jordan spend the most hours (and have the most inequitable distribution of hours across boys and girls) on domestic work. Likewise, this is the setting where we observe the largest number of gendered constraints on behaviour reported by girls, as well as the biggest difference in resilient coping scores between girls and boys, suggesting that the gendered expectations for adolescent behaviour exacerbated by the pandemic may impact multiple domains of wellbeing for girls in this setting. These findings support other research suggesting that the increased burden of domestic responsibilities for women and girls caused by the pandemic plays a role in the observed differences in measures of resilient coping.^2^^,^^3^^,^^9^^,^^59^

Our results further highlight both overarching trends and country-specific dynamics in adolescent responsibilities and time spent in the household across three diverse samples, identifying common protective factors for greater resilient coping (school enrolment), as well as common risk factors for adverse outcomes (underlying COVID-19 vulnerability, as well as child marriage and disability status). Somewhat unexpectedly, marital status emerged as a common protective factor for higher resilient coping scores on the BRCS-O. That said, married girls are likely to have already been living in more stressful conditions and to experience negative mental health outcomes and psychological distress compared to unmarried peers prior to the pandemic,^60^^,^^61^ thus the COVID-19 pandemic specific shock may have been more muted.

Critically, across settings, age, school enrolment, marital status, and underlying COVID-19 vulnerability were the strongest predictors of greater time spent on household responsibilities for boys and girls alike; girls have a higher burden of care and domestic work and hours on work tasks overall, while boys have a higher burden of engagement in agriculture and paid work across all three samples. Qualitative research on adolescent experiences in LMICs during the pandemic also supports this finding, as well as the particularly acute burden among girls who experienced child marriage.^27^ Within each country, location also served as a protective or risk factor. In rural areas of Ethiopia (East Hararghe and South Gondar) and Jordan (ITS communities), adolescents were significantly more likely to spend time on all work (domestic chores and agriculture or paid work) compared to adolescents living in other settings within the same country. This may reflect an underlying difference in the level of domestic and agricultural work that adolescents normally do in rural settings compared to urban settings prior to the pandemic, as well as the higher level of poverty in these rural areas compared to urban settings in both Ethiopia^62^ and Jordan.^29^

Within each setting, the differential impact of COVID-19 mitigation strategies employed by each country also plays a role in the trends observed. At the time of the survey (late 2020 and early 2021), pandemic-related restrictions were already being eased in Ethiopia, and Ethiopian students were already returning to school in both rural and urban areas, while schools in Jordan and Palestine would remain closed for several months. Because students were more likely to still be staying at home during the survey period in Jordan and Palestine, we may be more likely to observe differences in domestic work distribution and gendered constraints on girls’ behavior in the home based on public health measures, social restrictions, and school closures in these locations than in Ethiopia.

The strength of this study is that we present findings on the impact of the pandemic and its associated policies across three diverse contexts, with data collected using standardised instruments and procedures. We also adapted a set of adolescent-specific measures of wellbeing, following a rigorous process of creating and adapting valid social and behavioural measures on gender equality and empowerment,^22^^,^^23^ allowing us to better capture the impact of the pandemic on adolescents. In Jordan and Ethiopia, we were able to leverage longitudinal data to control for pre-pandemic differences in household characteristics associated with COVID-19 vulnerability.

However, there are some limitations to our analysis. First, it is cross-sectional, so findings should be interpreted as associations and not as causal. Second, our sample is a convenience sample, which limits generalisability. Additionally, while we completed a process to adapt survey measures in context, two key household responsibilities that are most common in low-income, rural settings were not included in the DWD module: time spent collecting water and time spent collecting fire wood/cooking fuel. This may lead to an underestimate of the amount of time adolescents and youth are truly spending on household responsibilities in rural Ethiopia, in particular, and may mask further gender differences for this population. Finally, our test-retest statistics suggest low measurement stability.

Ultimately, these documented short-term adverse effects of the pandemic on adolescent wellbeing are not only a concern in themselves, but might have longer-term repercussions. Reduced time and opportunity for social interaction with peers can hinder healthy adolescent development.^63^ Moreover, in households experiencing poverty and greater economic distress due to the pandemic, adolescents (particularly boys) are more likely to enter the workforce before adulthood.^27^^,^^64^^,^^65^ Furthermore, for girls, increased demands on time use for care work and other domestic tasks can combine with overall lower access to technology than male peers^66^ and pressure for girls to marry at an early age due to economic stressors,^3^ which all undermine girls’ ability to stay in (and succeed in) school.

Given the importance of young people for the prosperity of future generations,^67^ our findings highlight the need to focus attention on the most vulnerable, with greater attention to gender- and age-responsive social protection as an important policy tool to ensure an equitable post-pandemic recovery for adolescent boys and girls.^64^^,^^68^ In particular, given that household-level social protection does appear to be protective for the less vulnerable part of the study population (who themselves are still highly vulnerable) suggests that the most vulnerable need a greater level of economic support to buttress resilient coping.^69^ Moreover, because social protection support appears to be inadequate to offset restrictive gender norms, especially around girls’ time use, the findings also point to the importance of greater consideration of complementary or ‘plus’ measures that tackle not just economic but also social vulnerabilities as part of a comprehensive package of social protection support.^70^^,^^71^

## Contributors

SB, EO, and JS conceptualized this paper. Data curation was completed by SA, B Avuwadah, and EO. Formal analysis was completed by SB, EO, and JS. SB, NJ, and AM acquired the funding for this project. Investigation was carried out by B Abu Hamad, SA, JH, AM, JS, and WY. Methodology was created by SB. Writing of the original draft was completed by B Abu Hamad, SA, SB, EO, and JS. All authors contributed to review and editing (B Abu Hamad, SA, B Avuwadah, SB, JH, NJ, AM, EO, JS, and WY). All authors reviewed and edited the draft. All authors had full access to the data in the study and had final responsibility for the decision to submit for publication.

## Data sharing statement

De-identified, quantitative data from the GAGE study (and accompanying documentation) is made publicly available through the UK Data Archive. The quantitative data from the COVID-19 phone survey discussed in this paper is not yet available, but will be published to the UK Data Archive in 2022. Data for this analysis is available upon request from the authors.

## Declaration of interests

The authors declare no conflict of interest.

## Appendix Supplementary materials

Image, application 1

## Footnotes

1 For this analysis, we restrict to adolescents in the sample where surveys were completed for both the adolescent and the adult respondent and all covariates are available.

2 For Ethiopia, the BRCS was administered to a random subgroup of one-third of the total sample, resulting in a smaller number of respondents for these outcomes.

3 We exclude 13 observations where the total number of hours reported in either category (or both when summed) is greater than 24 hours. We acknowledge that it is possible for adolescents to engage in multiple activities at the same time (such as cleaning and child care), but we exclude these 13 adolescents who reported more than 24 hours of time spent on household responsibilities in the last day (24 hours) to avoid biasing the results.

4 There are two questions in this module where responses are restricted to a smaller sample; the question on menstrual hygiene management is only asked to girls who have begun menstruation, and the question on restrictions on being able to speak with family by phone is only asked to girls who are married. Accordingly, girls who have not reached menarche and who are not married have a maximum score of “5” on the 7-item scale.

5 In Ethiopia, selected assets used for this index include “TV or radio, fridge, or mattress”. In Jordan, selected assets include “fridge or fan/AC”, and in Palestine “TV, fridge or fan/AC”.

6 In Ethiopia, we consider location: Dire Dawa, Debre Tabor, South Gondar, and East Hararghe. In Jordan, covariates include nationality (Jordanian, Palestinian, Syrian, or other) and, among Syrian refugees only, location in Camp, Host community, or ITS. In Palestine, we consider location (West Bank or Gaza) and three groups related to refugee status: non-refugee, refugee living outside of a camp, and refugee living in a camp.

7 A *subkebele* is a small administrative unit in Ethiopia.
