# Supplementary material for: COVID-19 and the gendered impacts on adolescent wellbeing: Evidence from a cross-sectional study of locally adapted measures in Ethiopia, Jordan, and Palestine
Source: eClinicalMedicine. 2022 Aug 3;52:101586. doi: 10.1016/j.eclinm.2022.101586 (PMC9347265; doi:10.1016/j.eclinm.2022.101586)
Supplement: Supplementary file 1 [file mmc1.docx]

**Supplement to: COVID-19 and the gendered impacts on adolescent wellbeing: Evidence from a cross-section study of locally-adapted measures in Ethiopia, Jordan, and Palestine**

Erin Oakley^1^, Shoroq Abuhamad^2^, Jennifer Seager^1^, Benjamin Avuwadah^1^, Joan, Hamory^3^, Nicola Jones^4^, Agnieszka Małachowska^5^, Workneh Yadete^6^, Bassam Abu Hamad^2^, and Sarah Baird^1^

^1^ Department of Global Health, Milken Institute School of Public Health, George Washington University

^2^ Al-Quds University, Gaza, State of Palestine

^3^ Department of Economics, University of Oklahoma

^4^ Gender and Adolescence: Global Evidence (GAGE), and ODI, London, UK

^5^ Gender and Adolescence: Global Evidence (GAGE)

^6^ Gender and Adolescence: Global Evidence (GAGE) and Quest Consulting

**Supplementary Material Headings**

Appendix A. Measure development

Appendix B. Regression results

**Appendix A. Measure Development**

We present a summary of our work on measure development related to gendered impacts of COVID-19 on adolescent wellbeing in this appendix (Appendix A). The measures we developed are based on data from adapted survey modules for our population (adolescents) in all study settings (Ethiopia, Jordan, and Palestine). We began by selecting measures relevant to our study population from the EMERGE COVID-19 and Gender Survey Questions, compiled and adapted by the Center on Gender Equity and Health [1]. These initial modules included the following:

*Brief Resilient Coping Scale*

The Brief Resilient Coping Scale (BRCS) is a 4-item scale that measures “tendencies to cope with stress in a highly adaptive manner.” The BRCS was originally created by Sinclair and Wallston in 2004 [2], with an additional item related to the COVID-19 pandemic added by the EMERGE project.

*Gendered constraints on women and girls’ behaviors*

The gendered constraints on women and girls’ behavior scale (GCBS) was developed by the EMERGE project based on a literature review of relevant domains [3]. This series of five questions—asked only to female respondents—asks women and girls to consider the ways they have had to change their behavior at home “due to the increased presence of men in the household following the COVID-19 pandemic and the social containment efforts to manage the spread of the virus.”

*Domestic Work Distribution*

The Domestic Work Distribution (DWD) scale is adapted from the UN Women Rapid Assessment Survey on the socio-economic consequences of COVID-19 on women’s and men’s economic empowerment [4]. The EMERGE version of the scale asks respondents about six domestic and care work activities in the household and how time spent on these activities have changed following the pandemic.

Our work to further adapt the survey measures to our study population relied on the principles outlined in EMERGE guidelines for creating and adapting valid and reliable social and behavioral measures on gender equality and empowerment [5]. The work presented here starts with changes made to the survey modules based on feedback from first, the formative qualitative research, followed by the changes made based on cognitive interviews. We also provide results for the analysis of the psychometric properties of our measures in terms of their internal and external reliability, as applicable.

***Qualitative Interviews***

Formative qualitative research was the first step in our measure development process. The qualitative work consisted of in-depth interviews with adolescents and key informants for adolescent populations (such as healthcare workers, teachers, school counselors, NGO workers, government workers in family and social service fields, and male and female community leaders) in each setting. Thematic areas of focus for qualitative interviews across both adolescent interviews and key informant interviews included: adolescent coping strategies and resilience; intra-household gender and age divisions of labor; and intra-household relations during COVID-19-related shutdowns. We conducted in-depth interviews with 139 adolescents (60 in Ethiopia, 31 in Jordan, and 48 in Palestine) and 43 key informants (16 in Ethiopia, 10 in Jordan, and 17 in Palestine). The qualitative research team analyzed these interviews through a sequenced approach, beginning with an oral debriefing with the research teams from each country on preliminary findings, observations and key contextual variables, and then through thematic coding in MAQqda using a code book informed by the GAGE conceptual framework and the specific thematic foci related to the pandemic and associated public health impacts on gender and age-specific norms and role expectations.

Findings from this formative qualitative work informed the adaptations we made to the survey measures prior to cognitive interviews. Table A1 summarizes the key changes that stemmed from the qualitative interviews.

For additional context, we provide a more detailed illustrative example. Key informants in Jordan and Palestine, in particular, commented on the ways that men being in the home more often after pandemic-related closures and curfews led to challenges for adolescent girls beyond those that were originally included in the Gendered Constraints on Women’s/Girl’s Behavior module, such as increased demands on girls’ time in the household for meeting the needs of male relatives and supporting additional housework that may arise from having more family members staying at home. For example, one respondent—an NGO worker in Jordan—commented that girls were sometimes asked to cater to the needs of male relatives who were spending more time at home:

“Female [children] were pushed to stay wake the whole night to make coffee and tea for their male peers and their family.”

Based on these findings, we added the item “I have to spend more of my time responding to the demands of male household members because male members of the family are now home more” to the Gendered Constraints on Women’s/Girl’s Behavior module across settings.

**Table A1. Changes Implemented Based on Qualitative/Formative Research**

| **Original Module** | | **Adapted Module v1** | | **Explanatory Notes** |
| --- | --- | --- | --- | --- |
| **Module 1: Gendered Constraints on Women’s and Girl’s Behavior in the Home** | | | | |
| **Read:** In many households during the Corona lockdown, young women and adolescent girls have had to change the way they live in their own homes, due to increased presence of men in households during the day. Have you had to alter your behavior at home in any way due to the increased presence of men in the household following the Corona pandemic and the social containment efforts to manage the spread of the virus (lockdown, curfew, etc.)? Please respond yes or no to each of the following statements, as applies to you: | | **Read:** In many households during the Corona lockdown, young women and adolescent girls have had to change the way they live in their own homes, due to increased presence of men in households during the day. Have you had to alter your behavior at home in any way due to the increased presence of men in the household following the Corona pandemic and the social containment efforts to manage the spread of the virus (lockdown, curfew, etc.)? Please respond yes or no to each of the following statements, as applies to you: | | In this module, we added two new items based on the qualitative research and key informant interviews (6-7).  We also made minor changes to wording: removing the wording at the end of each item that said “and do not like me doing it” as this seemed unnecessary and would also leave out restrictions that we might care about (e.g., can’t watch tv because male members are always watching). |
| **1.** Since the start of the pandemic, I have to wear clothing that I would usually not wear while being at home, for example wearing only traditional clothes or clothes that cover more of my body.  ***[1=Yes, 2=No, -97=Refused]*** | | **1.** Since the start of the pandemic, I have to wear clothing that I would usually not wear while being at home, for example wearing only traditional clothes or clothes that cover more of my body.  ***[1=Yes, 2=No, -97=Refused]*** | |  |
| **2.** I cannot do things for entertainment that I used to do previously, for example watch my favorite TV show or listen to my favorite radio show, because male members of the family are now home and do not like me doing it.  ***[1=Yes, 2=No, -97=Refused]*** | | **2.** I cannot do things for entertainment that I used to do previously, for example watch my favorite TV show or listen to my favorite radio show, because male members of the family are now home.  ***[1=Yes, 2=No, -97=Refused]*** | |  |
| **3. Married adolescents only:** I cannot talk on the phone/chat online with my parents or other family members for as long as I used to previously, because male members of the family are now home and do not like me doing it.  ***[1=Yes, 2=No, -97=Refused]*** | | **3. Married adolescents only:** I cannot talk on the phone/chat online with my parents or other family members for as long as I used to previously, because male members of the family are now home.  ***[1=Yes, 2=No, -97=Refused]*** | |  |
| **4.** I cannot talk on the phone/chat online with my friends for as long as I used to previously, because male members of the family are now home and do not like me doing it.  ***[1=Yes, 2=No, -97=Refused]*** | | **4.** I cannot talk on the phone/chat online with my friends for as long as I used to previously, because male members of the family are now home.  ***[1=Yes, 2=No, -97=Refused]*** | |  |
| **5.** I cannot talk on the phone/chat online with male friends, because family members are home more and do not like me doing it.  ***[1=Yes, 2=No, -97=Refused]*** | | **5.** I cannot talk on the phone/chat online with male friends, because family members are home more.  ***[1=Yes, 2=No, -97=Refused]*** | |  |
|  | | **6. *Only if the adolescent confirms beginning menstruation:*** I find it more difficult to manage my menstruation cycle with brothers and fathers spending more time at home.  ***[1=Yes, 2=No, -97=Refused]*** | |  |
|  | | **7.** I have to spend more of my time responding to the demands of male household members because male members of the family are now home more.  ***[1=Yes, 2=No, -97=Refused]*** | |  |
| **Module 2: COVID-19 and Coping** | | | | |
| **8.** How well do each following statements describe your behavior and actions at this time?  ***After each statement, read response options aloud [Does not describe me at all; Does not describe me; Neutral; Describes me; Describes me very well]***  **Codes for Q8**  0= Does not describe me at all  1 = Does not describe me  2 = Neutral  3 = Describes me  4 = Describes me very well  ***Do not read aloud:*** *-*97=Refused | | **8.** How well do each following statements describe your behavior and actions at this time?  ***After each statement, read response options aloud [Does not describe me at all; Does not describe me; Neutral; Describes me; Describes me very well]***  **Codes for Q8**  0= Does not describe me at all  1 = Does not describe me  2 = Neutral  3 = Describes me  4 = Describes me very well  ***Do not read aloud:*** *-*97=Refused | | In this module, we added four additional items related to adolescent coping during the covid-19 pandemic based on findings from qualitative research. |
| **a.** I look for creative ways to alter difficult situations. | | **a.** I look for creative ways to alter difficult situations. | |  |
| **b.** Regardless of what happens to me, I believe I can control my reaction to it. | | **b.** Regardless of what happens to me, I believe I can control my reaction to it. | |  |
| **c.** I believe I can grow in positive ways by dealing with difficult situations. | | **c.** I believe I can grow in positive ways by dealing with difficult situations. | |  |
| **d.** I actively look for ways to replace the losses I encounter in life. | | **d.** I actively look for ways to replace the losses I encounter in life. | |  |
| **e.** I am coping well with the difficulty and stress caused by the Corona/COVID-19 pandemic. | | **e.** I am coping well with the difficulty and stress caused by the Corona/COVID-19 pandemic. | |  |
|  | | **f.** I seek comfort and guidance from religion. | |  |
|  | | **g.** My family is helping me cope with the difficulty and stress caused by the Corona/COVID-19 pandemic. | |  |
|  | | **h.** My friends are helping me cope with the difficulty and stress caused by the Corona/COVID-19 pandemic. | |  |
|  | | **i.** Adults (such as community members, sports coaches, or religious leaders) are helping me cope with the difficulty and stress caused by the Corona/COVID-19 pandemic | |  |
|  | | | | |
|  | | | | |
| **Module 3: Domestic Work Distribution** | | | | |
| ***Read:*** For each of the following, please indicate who has had to increase their time on these tasks since the COVID-19 lockdown, circle all that apply, and then check the person who takes greatest responsibility for this task. | | ***Read:*** Now I would like to ask you a few questions about domestic responsibilities in your home, including cooking, cleaning, and caring for family members. | | For the GAGE study, we are most interested in the experience of the adolescent, so we re-worded the main question to ask about whether his/her own tie spent on each activity has increased.  Then, instead of asking who does the most work, we ask specifically about how many hours they worked.  We also changed the wording of some items, added four additional items related to domestic and household responsibilities (f-i), and combined the original item c and item e into a single option, to reflect findings from qualitative research. |
| **9.** Compared to the time before the Corona pandemic and social restrictions in March 2020, who has increased work on this activity in the home?  ***[Read answer options aloud unless specified, select all that apply]*** | **10.** Today in your household, who does most of the work for this activity?  ***[Read answer options aloud unless specified, select only one]*** | **9.** Compared to the time before the Corona pandemic and social restrictions in March 2020, has the amount of time you spend on ***[ACTIVITY]*** increased, decreased, or stayed the same? | **10.** In the past 24 hours, how many hours did you spend doing ***[ACTIVITY]***?  ***[Round up to the nearest half hour;***  ***-97=Refused, -99=DK]*** |  |
| **Response Codes for Q9 and Q10:**  1=Mothers  2=Fathers  3=Daughters  4=Sons  5=Other family members  6=Someone outside of the family  ***Do not read:*** -99=DK, -97=Refused | | **Response Codes for Q9:**  1=Increased  2=Decreased  3=Stayed the same  4=I never do this activity, before or now  ***Do not read:*** -99=DK, -97=Refused | |  |
| **a.** House cleaning | | **a.** House cleaning, including disinfecting activities | |  |
| **b.** Cooking | | **b.** Cooking | |  |
| **c.** Taking care of children | | **c.** Taking care of children, older family members, or family members with a disability during normal times | |  |
| **d.** Taking care of family members when they are ill | | **d.** Taking care of family members when they are ill | |  |
| **e.** Taking care of older family members | |  | |  |
| **f.** Making sure the children are continuing to study and do schoolwork, or helping them with their studies | | **e.** Making sure siblings are continuing to study and do schoolwork, or helping them with their studies | |  |
|  | | **f.** Agricultural work for the household | |  |
|  |  | **g.** Working for the household business | |  |
|  |  | **h.** Non-household paid work | |  |
|  |  | **i.** Shopping for everyday supplies | |  |

Note: This table presents changes made to the original survey modules based on feedback from qualitative research. We present the original modules in the first column, followed by the adapted modules (changes made to the original module) in the second column and concise explanations for the changes we made in the last column. In the adapted module, items listed in light gray were re-worded based on findings from formative qualitative research. Items in dark gray are new questions or answer options emerging from the qualitative research that were not covered in the original set of questions.

***Cognitive Interviews***

After conducting formative qualitative research and incorporating an initial round of adaptations to the survey measures, we conducted a series of cognitive interviews in each setting, designed to assess whether adolescent respondents in the study setting correctly understood the content and response options for each question. Local enumerators followed a four-step process to assess respondent understanding of each question and gathered feedback on the question and response options. An excerpt from our four-step cognitive interviewing protocol is shown in Figure A1 below.

**Figure A1. Excerpt from Cognitive Interview Protocol – Ethiopia**

| For each measure, the enumerator should follow the following procedure:   1. Ask the question and have the respondent respond. 2. The enumerator should then ask the respondent how he/she understood the question and response options (if applicable), and note anything down relevant for further refining of measures and response options for clarity. 3. Enumerator should additionally ask about any words either in the question or the choice answers the respondent found difficult and note down these words 4. Enumerator should then rate whether they believe the respondent understand the question on a scale from 1-5 (1= Strongly Disagree, 2=Disagree, 3=Neither agree nor disagree, 4=Agree. 5=Strongly Agree). If any answer other than 4 or 5, should note why they feel this way |
| --- |

Cognitive Interviews were conducted within each sample with male and female adolescents of various ages and from different locations relevant to the sample. We also purposefully conducted interviews with adolescents from subpopulations of interest, including adolescents with disabilities, those who are out-of-school, and girls who experienced child marriage. Ultimately, we conducted the cognitive interviews for a total of 128 adolescents – 46 in Ethiopia, 62 in Jordan, and 20 in Palestine (see Table A2). Each interview took, on average: 25-30 minutes for boys age 14 and younger; 30-40 minutes for girls age 14 and younger; 70-80 minutes for boys age 15 and older; and 80-100 minutes for girls age 15 and older.

Following the cognitive interviews, local survey enumerators compiled the findings and feedback within each sample and proposed a series of updates to the survey tool to improve clarity and ensure the survey questions were appropriate and understandable for the survey population. Table A3 summarizes the suggested changes. Due to the similarities in language and, to some extent, context between Palestine and Jordan, we combined the proposed updates from Jordan and Palestine into a single common survey instrument with minimal differences between the two settings (Table A3, Panel A). While some similar changes were implemented in both the Jordan/Palestine and Ethiopia survey instruments, we considered the feedback on cognitive interviews separately for the Ethiopia instrument Table A3, Panel B).

As an illustrative example, researchers in Palestine noted the following points of feedback for the statement “I have to spend more of my time responding to the demands of male household members because male members of the family are now home more” in the Constraints on Women’s/Girl’s Behavior module:

“… [y]oung girls suggested to add ‘including the father’ to the statement, because many understood male family members include only the male siblings, excluding the father.”

Accordingly, we updated the language of this question for Palestine as follows: “I must fulfill the demands of males, including male siblings, fathers, or any other male family members, when they are at home more now since Corona started.”

**Table A2. Cognitive Interview Samples**

| ***Panel A: Ethiopia (n=46)*** | | | | | | | | | |
| --- | --- | --- | --- | --- | --- | --- | --- | --- | --- |
| Location | Younger Females | Younger Males | | Older Females (Unmarried, half out-of-school) | | Older Females  (Married) | Older Males  (In-school) | | Older Males  (Out-of-school) |
| East Haraghe | 2 | 2 | | 2 | | 1 | 0 | | 0 |
| South Gondar | 2 | 2 | | 2 | | 1 | 1 | | 1 |
| Debre Tabor | 2 | 2 | | 1 | | 3 | 1 | | 1 |
| Dire Dawa | 2 | 2 | | 1 | | 2 | 1 | | 1 |
| Adami Tulu | 0 | 0 | | 1 | | 3 | 1 | | 1 |
| Disability– one per location | 1 | 1 | | 1 | | 1 | 1* | | |
| ***Panel B: Jordan (n=62)*** | | | | | | | | | |
| Location/ Nationality | Younger Females | | Younger Males | | Older Females | | | Older Males | |
| Host (Syrian) | 2 | | 2 | | 2 | | | 2 | |
| Host (Jordanian) | 2 | | 2 | | 2 | | | 2 | |
| Informal Tent Settlement | 3 | | 3 | | 3 | | | 3 | |
| Azraq Camp | 2 | | 2 | | 2 | | | 2 | |
| Zaatari Camp | 2 | | 2 | | 2 | | | 2 | |
| Gaza Camp | 2 | | 2 | | 2 | | | 2 | |
| Disability (various locations/ nationality) | 1 | | 1 | | 1 | | | 1 | |
| Married (various locations/ nationality) | 0 | | 0 | | 6 | | | 0 | |
| ***Panel C: Palestine (Gaza only) (n=20)*** | | | | | | | | | |
| Sample | Younger Females | | Younger Males | | Older Females | | | Older Males | |
| General Sample | 3 | | 3 | | 4 | | | 3 | |
| Married | 0 | | 0 | | 3 | | | 0 | |
| Disability | 1 | | 1 | | 1 | | | 1 | |

Note: This table presents a breakdown of respondents for our cognitive interviews in each study setting. Across study settings, “Younger” adolescents are between the ages of 11-14, while “Older” adolescents are 15-21 years.

*The Ethiopia cognitive interview sample included five adolescents with disabilities, including one older male adolescent for whom school enrollment status was not pre-specified in the sampling plan.

**Table A3. Changes Implemented Based on Cognitive Interviews**

| **Adapted Module v1** | | **Final Survey Instrument** | | **Explanatory Notes** |
| --- | --- | --- | --- | --- |
| ***Panel A: Jordan and Palestine*** | | | | |
| **Module 1: Gendered Constraints on Women’s and Girl’s Behavior in the Home** | | | | |
| **Read:** In many households during the Corona lockdown, young women and adolescent girls have had to change the way they live in their own homes, due to increased presence of men in households during the day. Have you had to alter your behavior at home in any way due to the increased presence of men in the household following the Corona pandemic and the social containment efforts to manage the spread of the virus (lockdown, curfew, etc.)? Please respond yes or no to each of the following statements, as applies to you: | | **Read:** In many households during the Corona lockdown, young women and adolescent girls have had to change the way they live in their own homes, due to increased presence of men in households during the day. Have you had to alter your behavior at home in any way due to the increased presence of men in the household following the Corona pandemic and the social containment efforts to manage the spread of the virus (lockdown, curfew, etc.)? Please respond yes or no to each of the following statements, as applies to you: | | Based on feedback from cognitive interviews, we rephrased items 6 and 7 to improve understanding of the statements.  We also added an answer option for “I have no male friends” (item 5) based on feedback from cognitive interviews with adolescents. |
| **1.** Since the start of the pandemic, I have to wear clothing that I would usually not wear while being at home, for example wearing only traditional clothes or clothes that cover more of my body.  ***[1=Yes, 2=No, -97=Refused]*** | | **1.** Since the start of the pandemic, I have to wear clothing that I would usually not wear while being at home, for example wearing only traditional clothes or clothes that cover more of my body.  ***[1=Yes, 2=No, -97=Refused]*** | |  |
| **2.** I cannot do things for entertainment that I used to do previously, for example watch my favorite TV show or listen to my favorite radio show, because male members of the family are now home.  ***[1=Yes, 2=No, -97=Refused]*** | | **2.** I cannot do things for entertainment that I used to do previously, for example watch my favorite TV show or listen to my favorite radio show, because male members of the family are now home.  ***[1=Yes, 2=No, -97=Refused]*** | |  |
| **3. Married adolescents only:** I cannot talk on the phone/chat online with my parents or other family members for as long as I used to previously, because male members of the family are now home.  ***[1=Yes, 2=No, -97=Refused]*** | | **3. Married adolescents only:** I cannot talk on the phone/chat online with my parents or other family members for as long as I used to previously, because male members of the family are now home.  ***[1=Yes, 2=No, -97=Refused]*** | |  |
| **4.** I cannot talk on the phone/chat online with my friends for as long as I used to previously, because male members of the family are now home.  ***[1=Yes, 2=No, -97=Refused]*** | | **4.** I cannot talk on the phone/chat online with my friends for as long as I used to previously, because male members of the family are now home.  ***[1=Yes, 2=No, -97=Refused]*** | |  |
| **5.** I cannot talk on the phone/chat online with male friends, because family members are home more.  ***[1=Yes, 2=No, -97=Refused]*** | | **5.** I cannot talk on the phone/chat online with male friends, because family members are home more.  ***[1=Yes, 2=No, 3=I have no male friends, -97=Refused]*** | |  |
| **6. *Only if the adolescent confirms beginning menstruation:*** I find it more difficult to manage my menstruation cycle with brothers and fathers spending more time at home.  ***[1=Yes, 2=No, -97=Refused]*** | | **6.** ***Only if the CR confirms beginning menstruation:*** I struggle/ don't have freedom/ find difficulty when my period starts and my male family members are at home.  ***[1=Yes, 2=No, -97=Refused]*** | |  |
| **7.** I have to spend more of my time responding to the demands of male household members because male members of the family are now home more.  ***[1=Yes, 2=No, -97=Refused]*** | | **7.** I must fulfill the demands of males, including male siblings, fathers, or any other male family members, when they are at home more now since Corona started.  ***[1=Yes, 2=No, -97=Refused]*** | |  |
| **Module 2: COVID-19 and Coping** | | | | |
| **8.** How well do each following statements describe your behavior and actions at this time?  ***After each statement, read response options aloud [Does not describe me at all; Does not describe me; Neutral; Describes me; Describes me very well]***  **Codes for Q8**  0= Does not describe me at all  1 = Does not describe me  2 = Neutral  3 = Describes me  4 = Describes me very well  ***Do not read aloud:*** *-*97=Refused | | **8.** How well do each following statements describe your behavior and actions at this time?  ***After each statement, read response options aloud [Does not describe me at all; Does not describe me; Neutral; Describes me; Describes me very well]***  **Codes for Q8**  0= Does not describe me at all  1 = Does not describe me  2 = Neutral  3 = Describes me  4 = Describes me very well  ***Do not read aloud:*** *-*97=Refused | | In the final, locally-adapted measures, we incorporated several changes to the wording of the original scale to improve adolescent understanding of the statement based on feedback from the cognitive interview process (items a-d).  We also reworded new survey item h to improve understanding.  Additionally, we incorporated more examples into items f and i. |
| **a.** I look for creative ways to alter difficult situations. | | **a.** I try to find/ come up with innovative/new ways to face difficult situations. | |  |
| **b.** Regardless of what happens to me, I believe I can control my reaction to it. | | **b.** No matter what happens to me, I control my reaction to what is happening. | |  |
| **c.** I believe I can grow in positive ways by dealing with difficult situations. | | **c.** I believe that I can develop myself in positive ways by dealing with difficult situations. | |  |
| **d.** I actively look for ways to replace the losses I encounter in life. | | **d.** I am eagerly searching for ways to recoup the losses I am facing in life | |  |
| **e.** I am coping well with the difficulty and stress caused by the Corona/COVID-19 pandemic. | | **e.** I am coping well with the difficulty and stress caused by the Corona/COVID-19 pandemic. | |  |
| **f.** I seek comfort and guidance from religion. | | **f.** I seek comfort and guidance from religion, such as by praying, reading from the holy book, or other religious activities | |  |
| **g.** My family is helping me cope with the difficulty and stress caused by the Corona/COVID-19 pandemic. | | **g.** My family is helping me cope with the difficulty and stress caused by the Corona/COVID-19 pandemic. | |  |
| **h.** My friends are helping me cope with the difficulty and stress caused by the Corona/COVID-19 pandemic. | | **h.** My friends offer me help and assistance to overcome the difficulty and stress caused by Corona | |  |
| **i.** Adults (such as community members, sports coaches, or religious leaders) are helping me cope with the difficulty and stress caused by the Corona/COVID-19 pandemic | | **i.** Adults (such as community members, sports coaches, religious leaders, or teachers) are helping me cope with the difficulty and stress caused by the Corona/COVID-19 pandemic | |  |
|  | | | | |
|  | | | | |
| **Module 3: Domestic Work Distribution** | | | | |
| ***Read:*** Now I would like to ask you a few questions about domestic responsibilities in your home, including cooking, cleaning, and caring for family members. | | ***Read:*** Now I would like to ask you a few questions about domestic responsibilities in your home, including cooking, cleaning, and caring for family members. | | Minor updates to the Domestic Work Distribution module include rephrasing item h and adding a new item mentioned by adolescents related to work completed at home during the pandemic (item j). |
| **9.** Compared to the time before the Corona pandemic and social restrictions in March 2020, has the amount of time you spend on ***[ACTIVITY]*** increased, decreased, or stayed the same? | **10.** In the past 24 hours, how many hours did you spend doing ***[ACTIVITY]***?  ***[Round up to the nearest half hour;***  ***-97=Refused, -99=DK]*** | **9.** Compared to the time before the Corona pandemic and social restrictions in March 2020, has the amount of time you spend on ***[ACTIVITY]*** increased, decreased, or stayed the same? | **10.** In the past 24 hours, how many hours did you spend doing ***[ACTIVITY]***?  ***[Round up to the nearest half hour;***  ***-97=Refused, -99=DK]*** |  |
| **Response Codes for Q9:**  1=Increased  2=Decreased  3=Stayed the same  4=I never do this activity, before or now  ***Do not read:*** -99=DK, -97=Refused | | **Response Codes for Q9:**  1=Increased  2=Decreased  3=Stayed the same  4=I never do this activity, before or now  ***Do not read:*** -99=DK, -97=Refused | |  |
| **a.** House cleaning, including disinfecting activities | | **a.** House cleaning, including disinfecting activities | |  |
| **b.** Cooking | | **b.** Cooking | |  |
| **c.** Taking care of children, older family members, or family members with a disability during normal times | | **c.** Taking care of children, older family members, or family members with a disability during normal times | |  |
| **d.** Taking care of family members when they are ill | | **d.** Taking care of family members when they are ill | |  |
| **e.** Making sure siblings are continuing to study and do schoolwork, or helping them with their studies | | **e.** Making sure siblings are continuing to study and do schoolwork, or helping them with their studies | |  |
| **f.** Agricultural work for the household | | **f.** Agricultural work for the household | |  |
| **g.** Working for the household business | | **g.** Working for the household business | |  |
| **h.** Non-household paid work | | **h.** Paid work outside the household | |  |
| **i.** Shopping for everyday supplies | | **i.** Shopping for everyday supplies | |  |
|  | | **j.** Paid work inside the home (such as embroidery, food preparation, or other work that is done from home) | |  |

| **Adapted Module v1** | | **Final Survey Instrument** | | **Explanatory Notes** |
| --- | --- | --- | --- | --- |
| ***Panel B: Ethiopia*** | | | | |
| **Module 1: Gendered Constraints on Women’s and Girl’s Behavior in the Home** | | | | |
| **Read:** In many households during the Corona lockdown, young women and adolescent girls have had to change the way they live in their own homes, due to increased presence of men in households during the day. Have you had to alter your behavior at home in any way due to the increased presence of men in the household following the Corona pandemic and the social containment efforts to manage the spread of the virus (lockdown, curfew, etc.)? Please respond yes or no to each of the following statements, as applies to you: | | ***Read:*** In many households during the Corona lockdown, young women and adolescent girls have had to change the way they live in their own homes, due to increased presence of men in households during the day. Have you had to alter your behavior at home in any way due to the increased presence of men in the household following the Corona pandemic and the social containment efforts to manage the spread of the virus (lockdown, curfew, etc.)? Please respond yes or no to each of the following statements, as applies to you: | | The only change we made to this module was adding an answer option “I have no male friends” to item **5**, based on feedback from the cognitive interviews. |
| **1.** Since the start of the pandemic, I have to wear clothing that I would usually not wear while being at home, for example wearing only traditional clothes or clothes that cover more of my body.  ***[1=Yes, 2=No, -97=Refused]*** | | 1. Since the start of the pandemic, I have to wear clothing that I would usually not wear while being at home, for example wearing only traditional clothes or clothes that cover more of my body.  ***[1=Yes, 2=No, -97=Refused]*** | |  |
| **2.** I cannot do things for entertainment that I used to do previously, for example watch my favorite TV show or listen to my favorite radio show, because male members of the family are now home.  ***[1=Yes, 2=No, -97=Refused]*** | | 2. I cannot do things for entertainment that I used to do previously, for example watch my favorite TV show or listen to my favorite radio show, because male members of the family are now home.  ***[1=Yes, 2=No, -97=Refused]*** | |  |
| **3. Married adolescents only:** I cannot talk on the phone/chat online with my parents or other family members for as long as I used to previously, because male members of the family are now home.  ***[1=Yes, 2=No, -97=Refused]*** | | ***3. Married adolescents only:***  I cannot talk on the phone/chat online with my parents or other family members for as long as I used to previously, because male members of the family are now home.  ***[1=Yes, 2=No, -97=Refused]*** | |  |
| **4.** I cannot talk on the phone/chat online with my friends for as long as I used to previously, because male members of the family are now home.  ***[1=Yes, 2=No, -97=Refused]*** | | 4. I cannot talk on the phone/chat online with my friends for as long as I used to previously, because male members of the family are now home.  ***[1=Yes, 2=No, -97=Refused]*** | |  |
| **5.** I cannot talk on the phone/chat online with male friends, because family members are home more.  ***[1=Yes, 2=No, -97=Refused]*** | | **5**. I cannot talk on the phone/chat online with male friends, because family members are home more.  ***[1=Yes, 2=No, 3=I have no male friends, -97=Refused]*** | |  |
| **6. *Only if the adolescent confirms beginning menstruation:*** I find it more difficult to manage my menstruation cycle with brothers and fathers spending more time at home.  ***[1=Yes, 2=No, -97=Refused]*** | | ***6. Only if the adolescent confirms beginning menstruation***: I find it more difficult to manage my menstruation cycle with male family members spending more time at home.  ***[1=Yes, 2=No, -97=Refused]*** | |  |
| **7.** I have to spend more of my time responding to the demands of male household members because male members of the family are now home more.  ***[1=Yes, 2=No, -97=Refused]*** | | **7.** I have to spend more of my time responding to the demands of male household members because male members of the family are now home more.  ***[1=Yes, 2=No, -97=Refused]*** | |  |
| **Module 2: COVID-19 and Coping** | | | | |
| **8.** How well do each following statements describe your behavior and actions at this time?  ***After each statement, read response options aloud [Does not describe me at all; Does not describe me; Neutral; Describes me; Describes me very well]***  **Codes for Q8**  0= Does not describe me at all  1 = Does not describe me  2 = Neutral  3 = Describes me  4 = Describes me very well  ***Do not read aloud:*** *-*97=Refused | | **8.** How well do each following statements describe your behavior and actions at this time?  ***After each statement, read response options aloud [Does not describe me at all; Does not describe me; Neutral; Describes me; Describes me very well]***  **Codes for Q8**  0= Does not describe me at all  1 = Does not describe me  2 = Neutral  3 = Describes me  4 = Describes me very well  ***Do not read aloud:*** *-*97=Refused | | We made two main adaptations: first, the wording was adapted on the first item to better convey the concept of creativity.  Second, unlike the instrument for Jordan/Palestine, we changed “Corona/COVID-19 pandemic” in items **e**, **g** – **i** to “Coronavirus pandemic”, which was more appropriate to the local context. |
| **a.** I look for creative ways to alter difficult situations. | | **a.** I try to find/ come up with innovative/new ways to face difficult situations. | |  |
| **b.** Regardless of what happens to me, I believe I can control my reaction to it. | | **b.** Regardless of what happens to me, I believe I can control my reaction to it. | |  |
| **c.** I believe I can grow in positive ways by dealing with difficult situations. | | **c.** I believe I can grow in positive ways by dealing with difficult situations. | |  |
| **d.** I actively look for ways to replace the losses I encounter in life. | | **d.** I actively look for ways to replace the losses I encounter in life. | |  |
| **e.** I am coping well with the difficulty and stress caused by the Corona/COVID-19 pandemic. | | **e.** I am coping well with the difficulty and stress caused by the Coronavirus pandemic. | |  |
| **f.** I seek comfort and guidance from religion. | | **f.** I seek comfort and guidance from religion. | |  |
| **g.** My family is helping me cope with the difficulty and stress caused by the Corona/COVID-19 pandemic. | | **g.** My family is helping me cope with the difficulty and stress caused by the Coronavirus pandemic. | |  |
| **h.** My friends are helping me cope with the difficulty and stress caused by the Corona/COVID-19 pandemic. | | **h.** My friends are helping me cope with the difficulty and stress caused by the Coronavirus pandemic. | |  |
| **i.** Adults (such as community members, sports coaches, or religious leaders) are helping me cope with the difficulty and stress caused by the Corona/COVID-19 pandemic | | **i.** Adults (such as community members, sports coaches, or religious leaders) are helping me cope with the difficulty and stress caused by the Coronavirus pandemic | |  |
|  | | | | |
|  | | | | |
| **Module 3: Domestic Work Distribution** | | | | |
| ***Read:*** Now I would like to ask you a few questions about domestic responsibilities in your home, including cooking, cleaning, and caring for family members. | | ***Read:*** Now I would like to ask you a few questions about domestic responsibilities in your home, including cooking, cleaning, and caring for family members. | | For this set of questions, we added item **j** to capture adolescents work at home during the pandemic. |
| **9.** Compared to the time before the Corona pandemic and social restrictions in March 2020, has the amount of time you spend on ***[ACTIVITY]*** increased, decreased, or stayed the same? | **10.** In the past 24 hours, how many hours did you spend doing ***[ACTIVITY]***?  ***[Round up to the nearest half hour;***  ***-97=Refused, -99=DK]*** | **9.** Compared to the time before early Megabit, has the amount of time you spend on [ACTIVITY] increased, decreased, or stayed the same? | **10.** In the past 24 hours, how many hours did you spend doing ***[ACTIVITY]***? ***[Round up to the nearest half hour, -97=Refused, -99=DK]*** |  |
| **Response Codes for Q9:**  1=Increased  2=Decreased  3=Stayed the same  4=I never do this activity, before or now  ***Do not read:*** -99=DK, -97=Refused | | **Response Codes:** 1=Increased 2=Decreased 3=Stayed the same 4=I never do this activity, before or now ***Do not read:*** -99=DK, -97=Refused | |  |
| **a.** House cleaning, including disinfecting activities | | **a.** House cleaning, including disinfecting activities | |  |
| **b.** Cooking | | **b.** Cooking | |  |
| **c.** Taking care of children, older family members, or family members with a disability during normal times | | **c.** Taking care of children, older family members, or family members with a disability during normal times | |  |
| **d.** Taking care of family members when they are ill | | **d.** Taking care of family members when they are ill | |  |
| **e.** Making sure siblings are continuing to study and do schoolwork, or helping them with their studies | | **e.** Making sure siblings are continuing to study and do schoolwork, or helping them with their studies | |  |
| **f.** Agricultural work for the household | | **f.** Agricultural work for the household | |  |
| **g.** Working for the household business | | **g.** Working for the household business | |  |
| **h.** Non-household paid work | | **h.** Non-household paid work | |  |
| **i.** Shopping for everyday supplies | | **i.** Shopping for everyday supplies | |  |
|  | | **j.** Paid work inside the home (embroidery, food preparation, or other work done from home) | |  |

Note: This table presents changes made to the adapted survey modules based on feedback from our cognitive interview research. We present the adapted modules in the first column, followed by the final survey modules (changes made to the adapted module) in the second column and concise explanations for the changes we made in the last column. In the final module, items listed in light gray were re-worded based on feedback from cognitive interviews. Items in dark gray are new questions or answer options emerging from the cognitive interview process that were not covered in the first version of the adapted module.

***Psychometrics***

*Internal Reliability*

We use Cronbach’s alpha to assess the internal reliability for the two survey modules that include produce an outcome scored on a continuous scale and where we expected to find significant levels of inter-item correlation: the adapted BRCS and the GCBS. All tests of internal reliability were conducted within our full sample of 5,752 adolescents.

For the adapted BRCS module, we calculated Cronbach’s alpha for the full sample and within each setting (Ethiopia, Jordan, Palestine) for the full nine-item scale (BRCS-Total) as well as separately for the original four-item instrument BRCS-O and the five-item BRCS-COVID-19 (see Table A4). For each of the three coping scales, Cronbach’s alpha suggests moderate to acceptable internal reliability [6]. Overall, alpha for the original, four-item BRCS is similar to that calculated by Sinclair and Wallston in their original study, which ranged from 0.64 to 0.76 for different samples and time points [2].

The seven-item GCBS showed acceptable internal reliability based on Cronbach’s alpha (0.80 across all samples), ranging from a high of 0.82 in Ethiopia to a low of 0.75 in Jordan.

*External Reliability and Stability*

We conducted a retest of the three modules of interest for a small subset of adolescents in Ethiopia (n=180) and Jordan (n=64) to examine the external reliability and stability of the survey measures over time. Because the BRCS-O and BRCS-COVID-19 were administered to a random subgroup of one third of the full sample of adolescents in Ethiopia, the retest sample size for this survey module in Ethiopia is smaller (n=44). On average, retests were administered 4.2 weeks after the initial survey in Jordan and 2.9 weeks after the initial survey in Ethiopia; however, time between the initial survey administration and follow-up ranged from 2 to 7 weeks in Jordan and 1 weeks to 10 weeks in Ethiopia, potentially contributing to the change observed across rounds of survey administration.

For the survey modules scored on a continuous scale where stability is particularly of interest (BRCS-Total, BRCS-O, BRCS-COVID-19, and GCBS), we calculate two measures of external reliability: Intraclass Correlation Coefficient (ICC) using a two-way, mixed effects model to assess the level of absolute agreement in scores between the two rounds [7,8,9] and Pearson’s r to assess the correlation in scores between the two rounds [5].

Across both tests, we find poor test-retest reliability for the BRCS-O, BRCS-COVID-19, and BRCS-Total in Ethiopia and Jordan (see Table A5). Agreement is slightly higher in both settings for the BRCS-O, with ICC 0.23 (95% CI: -0.02, 0.45) in Jordan and ICC 0.13 (-0.18, 0.41). Correlation coefficients are similarly low for the BRCS-O in both settings, although the correlation is more significant in Jordan (r=0.23, p=0.08) than Ethiopia (r=0.13, p=0.40). Overall, the BRCS-Total does not demonstrate stability over the time period between surveys in our study, with an ICC of 0.21 (95% CI: -0.04, 0.43) in Jordan and 0.10 (95% CI: -0.19, 0.38) in Ethiopia.

For the GCBS, we find higher test-retest reliability in Jordan, where the ICC is 0.46 (95% CI: 0.15, 0.69). However, this is still below levels generally considered to be acceptable to establish test-retest reliability [8,9]. In Ethiopia, we find no agreement, with a negative correlation coefficient (r= -0.227, p=0.074) and negative ICC (-0.201, 95% confidence interval: -0.418, 0.041). This may reflect the low mean of the scores for this scale in Ethiopia; within the Ethiopian test-retest sample (n=71), adolescents scored a mean of 0.87 (standard deviation 1.36) at the initial survey and 1.39 (standard deviation 1.92) during the second administration.

For the DWD module, we do not expect to see high levels of absolute agreement in questions between the test and retest administration because the DWD module deals with time use in the last 24 hours and perceived change in time use currently compared to before the pandemic. However, it is still of interest to examine the relative stability of these measures over time to determine how well time use responses at the first administration predict time use responses in the near future.

First, we examined the stability of categorical questions in the DWD module (i.e., those focused on perceived change in time use compared to before the pandemic) using Cohen’s kappa [10,11]. In Jordan, stability was fair to moderate for perceived change in time spent on all household responsibilities due to the pandemic, except for time spent working in a household business and time spent shopping for everyday supplies (Table A6). In Ethiopia, stability was fair to moderate for most tasks, including cooking, cleaning, child and elder care, agricultural work, and working for a household business.

Next, we examined change in responses for the retest sample for the questions on number of hours spent on various household tasks. Because day-to-day time requirements are likely highly variable for adolescent domestic responsibilities, we do not expect to see high levels of absolute agreement in these responses. As such, we only calculate the correlation of responses between rounds. These results are presented in Table A7. Notably, correlation was high for the number of hours spent on tasks such as paid work outside the household (r=0.66, p<0.001), cleaning (r=0.49, p<0.001), and child or elder care (r=0.45, p<0.001) in Jordan, and working in a household business (r=0.54, p<0.001), cooking (r=0.46, p<0.001), and agricultural work for the household (r=0.40, p<0.001) in Ethiopia.

**Table A4. Cronbach’s alpha for relevant survey measures**

| **Survey Measure** | **All Samples** | **Jordan** | **Palestine** | **Ethiopia*** |
| --- | --- | --- | --- | --- |
| BRCS-O (0-16) | 0.681 | 0.631 | 0.785 | 0.734 |
| BRCS-COVID-19 (0-20) | 0.639 | 0.573 | 0.672 | 0.762 |
| BRCS-Total (0-36) | 0.743 | 0.704 | 0.816 | 0.788 |
| GCBS (0-7) | 0.802 | 0.746 | 0.783 | 0.816 |

Notes: This table presents Cronbach’s alpha for the BRCS-O, the BRCS-COVID-19, the total nine-item BRCS module (BRCS-Total), and the GCBS. The total sample size is 4,582 for the BRCS-O; 4,584 for the BRCS-COVID-19 and the BRCS-Total; and 2,864 for the GCBS, which is only administered to adolescent girls.

*The BRCS-O, BRCS-COVID-19, and BRCS-Total were administered to a randomly-assigned subgroup of one third of the full sample of adolescents in Ethiopia.

**Table A5. Test-retest reliability for survey measures scored on a continuous scale**

| Survey Measure | Jordan | | | Ethiopia* | | |
| --- | --- | --- | --- | --- | --- | --- |
|  | ICC (95% CI) | r (p) | n | ICC (95% CI) | r (p) | n |
| BRCS-O (0-16) | 0.23 (-0.02, 0.45) | 0.23 (0.08) | 63 | 0.13 (-0.18, 0.41) | 0.13 (0.4) | 44 |
| BRCS-COVID-19 (0-20) | 0.21 (-0.04, 0.43) | 0.21 (0.1) | 63 | -0.07 (-0.35, 0.23) | -0.07 (0.64) | 44 |
| BRCS-Total (0-36) | 0.21 (-0.05, 0.43) | 0.21 (0.1) | 62 | 0.1 (-0.19, 0.38) | 0.1 (0.5) | 44 |
| GCBS (0-7) | 0.46 (0.15, 0.69) | 0.46 (0.01) | 35 | -0.2 (-0.42, 0.04) | -0.17 (0.15) | 71 |

Notes: This table presents the Intraclass Correlation Coefficient (ICC) with 95% confidence intervals, the correlation coefficient with p-value, and sample size for each test-retest survey module in Jordan and Ethiopia. The ICC is calculated using a two-way, mixed effects model for absolute agreement; we present the individual result (rather than average). The correlation coefficient is calculated using Pearson’s r. The GCBS is administered only to adolescent girls.

*The BRCS-O, BRCS-COVID-19, and BRCS-Total were administered to a randomly-assigned subgroup of one third of the full sample of adolescents in Ethiopia.

**Table A6. Test-retest reliability for categorical questions in DWD module**

| Household Activity | Jordan | | Ethiopia | |
| --- | --- | --- | --- | --- |
|  | Kappa | n | Kappa | n |
| a. House cleaning, including disinfecting activities | 0.295 | 63 | 0.296 | 180 |
| b. Cooking | 0.367 | 63 | 0.431 | 180 |
| c. Taking care of children, older family members, or family members with a disability during normal times | 0.208 | 63 | 0.251 | 180 |
| d. Taking care of family members when they are ill | 0.272 | 63 | 0.183 | 180 |
| e. Making sure siblings are continuing to study and do schoolwork, or helping them with their studies | 0.465 | 63 | 0.159 | 180 |
| f. Agricultural work for the household | 0.259 | 63 | 0.529 | 180 |
| g. Working for the household business | -0.012 | 63 | 0.289 | 180 |
| h. Paid work outside the household /  h. Non-household paid work | 0.457 | 63 | 0.150 | 180 |
| i. Shopping for everyday supplies | 0.075 | 63 | 0.042 | 180 |
| j. Paid work inside the home (such as embroidery, food preparation, or other work that is done from home) | n/a* | 37 | -0.0283 | 180 |

Notes: This table presents the Kappa statistic and sample size for the test-retest analysis of categorical questions in the DWD module (i.e., for the question: “Compared to the time before the Corona pandemic and social restrictions in March 2020, has the amount of time you spend on *[ACTIVITY]* increased, decreased, or stayed the same?”).

*This estimate is not available for Jordan due to an insufficient number of observations with heterogenous responses.

**Table A7. Test-retest correlation for continuous questions in DWD module**

| Household Activity | Jordan | | Ethiopia | |
| --- | --- | --- | --- | --- |
|  | r (p) | n | r (p) | n |
| a. House cleaning, including disinfecting activities | 0.49 (<0.001) | 63 | 0.27 (<0.001) | 180 |
| b. Cooking | 0.25 (0.05) | 63 | 0.46 (<0.001) | 180 |
| c. Taking care of children, older family members, or family members with a disability during normal times | 0.45 (<0.001) | 63 | 0.22 (<0.001) | 180 |
| d. Taking care of family members when they are ill | 0.04 (0.75) | 63 | 0.24 (<0.001) | 180 |
| e. Making sure siblings are continuing to study and do schoolwork, or helping them with their studies | 0.27 (0.03) | 63 | 0.21 (<0.001) | 180 |
| f. Agricultural work for the household | -0.01 (0.92) | 63 | 0.4 (<0.001) | 180 |
| g. Working for the household business | n/a | 63 | 0.54 (<0.001) | 180 |
| h. Paid work outside the household /  h. Non-household paid work | 0.66 (<0.001) | 63 | -0.02 (0.8) | 180 |
| i. Shopping for everyday supplies | -0.02 (0.86) | 63 | 0.02 (0.78) | 180 |
| j. Paid work inside the home (such as embroidery, food preparation, or other work that is done from home) | n/a | 37 | -0.02 (0.77) | 180 |

Notes: This table presents correlation coefficient (with p-value) and sample size for the test-retest analysis of continuous questions in the DWD module (i.e., for the question: “In the past 24 hours, how many hours did you spend doing *[ACTIVITY]*?”). The correlation coefficient is calculated using Pearson’s r.

*This estimate is not available for Jordan due to an insufficient number of observations with an answer above “0”.

**References for Appendix A. Measure Development**

1. Center on Gender Equity and Health (GEH). EMERGE COVID-19 and Gender Survey Questions; 2020. <https://emerge.ucsd.edu/covid-19/>
2. Sinclair, V.G., Wallston, K.A. The development and psychometric evaluation of the Brief Resilient Coping Scale. Assessment. 2004; 11(1): 94-101. <https://pubmed.ncbi.nlm.nih.gov/14994958/>
3. Center on Gender Equity and Health (GEH). EMERGE COVID-19 and Gender Questions: Women and Girls’ Agency; 2020. <https://emerge.ucsd.edu/wp-content/uploads/2020/07/emerge-covid-and-gender-questions-women-and-girls-agency.pdf>
4. UN Women. Survey shows that COVID-19 has gendered effects in Asia and the Pacific. UN Women: Women Count; 2020. <https://data.unwomen.org/resources/surveys-show-covid-19-has-gendered-effects-asia-and-pacific>
5. Jose, R., Bhan, N., & Raj, A. EMERGE Measurement Guidelines Report 2: How to Create Scientifically Valid Social and Behavioral Measures on Gender Equality and Empowerment. Center on Gender Equity and Health (GEH), University of California, San Diego School of Medicine. San Diego, CA. November, 2017.
6. Tavakol, M. and Dennick, R. Making sense of Cronbach’s alpha. *Int J Med Educ.* 2011; 2: 53-55. <https://www.ncbi.nlm.nih.gov/pmc/articles/PMC4205511/#r11>
7. McGraw, Kenneth O., Wong, S.P. Forming Inferences About Some Intraclass Correlation Coefficients. Psychological methods, 1996-03, Vol.1 (1), p.30-46. DOI: 10.1037/1082-989X.1.1.30
8. Aldridge, V.K., Dovey, T.M., Wade, A. Assessing test-retest reliability of psychological measures. European Psychologist 2017; 22(4): 207-218. <https://doi.org/10.1027/1016-9040/a000298>
9. Koo, T.K., Li, M.Y. A guideline of selecting and reporting Intraclass Correlation Coefficients for Reliability Research. *J Chiropr Med.* 2016; 15(2): 155-163. <https://www.ncbi.nlm.nih.gov/pmc/articles/PMC4913118/#bb0010>
10. Cohen, J. A coefficient of agreement for nominal scales. Educational and Psychological Measurement. 1960; 20(1): 37-46. <https://doi.org/10.1177/001316446002000104>
11. McHugh, M.L. Interrater reliability: the kappa statistic. Biochem Med. 2012; 22(3): 276-282. <https://www.ncbi.nlm.nih.gov/pmc/articles/PMC3900052/>

**Appendix B. Regression results**

T**able B1. Covariate coefficients, whole sample (boys and girls in Ethiopia, Jordan, Palestine)**

|  | (1) | (2) | (3) | (4) | (5) |
| --- | --- | --- | --- | --- | --- |
|  | BRCS-O | BRCS-COVID-19 | Domestic Work | Ag/Paid Work | Domestic or Ag/Paid Work |
| Female | -0.073** | -0.048 | 0.569*** | -0.445*** | 0.185*** |
|  | (0.031) | (0.030) | (0.026) | (0.027) | (0.028) |
| Age | 0.053*** | 0.011 | 0.017*** | 0.035*** | 0.036*** |
|  | (0.007) | (0.007) | (0.006) | (0.006) | (0.006) |
| Above median on asset index | 0.032 | 0.098*** | -0.055* | -0.017 | -0.057* |
|  | (0.034) | (0.034) | (0.030) | (0.030) | (0.031) |
| Ever Married | 0.143** | 0.032 | 0.582*** | -0.160*** | 0.390*** |
|  | (0.065) | (0.065) | (0.068) | (0.062) | (0.064) |
| Disability status | -0.120** | -0.093** | -0.038 | -0.112*** | -0.100** |
|  | (0.048) | (0.046) | (0.042) | (0.037) | (0.042) |
| Enrolled in school March 2020 | 0.248*** | 0.130*** | -0.001 | -0.295*** | -0.177*** |
|  | (0.046) | (0.046) | (0.039) | (0.052) | (0.044) |
| Eth: South Gondor (rural) | -0.082 | -0.030 | 0.121* | 0.594*** | 0.462*** |
|  | (0.085) | (0.083) | (0.067) | (0.063) | (0.067) |
| Eth: East Haraghe (rural) | 0.214** | 0.320*** | 0.132* | 0.083 | 0.155** |
|  | (0.090) | (0.098) | (0.068) | (0.060) | (0.067) |
| Eth: Debre Tabor (urban) | -0.132* | 0.063 | 0.099 | -0.191*** | -0.038 |
|  | (0.075) | (0.082) | (0.070) | (0.059) | (0.067) |
| Eth: Dire Dawa (urban) | 0.182** | 0.423*** | -0.084 | -0.247*** | -0.209*** |
|  | (0.077) | (0.091) | (0.073) | (0.064) | (0.068) |
| Jord: Syrian refugee living in host community | 0.140** | 0.210*** | 0.096 | 0.026 | 0.092 |
|  | (0.065) | (0.065) | (0.062) | (0.054) | (0.062) |
| Jord: Syrian refugee living in camp | 0.146** | 0.367*** | 0.093 | -0.048 | 0.048 |
|  | (0.073) | (0.072) | (0.067) | (0.059) | (0.067) |
| Jord: Syrian refugee living in ITS | -0.046 | 0.409*** | 0.235*** | 0.089 | 0.255*** |
|  | (0.097) | (0.094) | (0.089) | (0.101) | (0.096) |
| Jord: Palestinian refugee | -0.163* | 0.005 | 0.149 | 0.066 | 0.167* |
|  | (0.090) | (0.095) | (0.091) | (0.081) | (0.091) |
| Jord: Other nationality | 0.109 | 0.120 | -0.092 | -0.296*** | -0.255* |
|  | (0.148) | (0.160) | (0.150) | (0.090) | (0.135) |
| Pal: Gaza | 0.030 | 0.446*** | 0.423*** | 0.067 | 0.403*** |
|  | (0.089) | (0.085) | (0.088) | (0.072) | (0.087) |
| Pal: West Bank | 0.348*** | 0.463*** | -0.098 | 0.173** | 0.019 |
|  | (0.070) | (0.071) | (0.064) | (0.077) | (0.071) |
| Pal: Lives in a refugee camp | -0.382*** | -0.716*** | -0.206*** | -0.195*** | -0.298*** |
|  | (0.097) | (0.087) | (0.063) | (0.062) | (0.066) |
| Pal: Refugee not living in a camp | 0.028 | -0.199** | -0.122 | -0.115 | -0.177* |
|  | (0.086) | (0.090) | (0.096) | (0.086) | (0.096) |
| Number of observations | 4,577 | 4,581 | 5,700 | 5,734 | 5,700 |

*Notes.*  Each panel of the table presents separate specification. Each column is a separate regression of the outcome specified in the header on the covariates listed in the rows. Eth=Ethiopia, Jord=Jordan, Pal=Palestine. See Table 1 for notes on covariates. Standard errors are clustered at the *subkebele* level in Ethiopia to account for sampling design, and at the individual level in Jordan and Palestine and presented in parentheses. *** p<.01, ** p<.05, * p<.10

T**able B2. Covariate coefficients, girls only (Ethiopia, Jordan, Palestine)**

|  | (1) | (2) | (3) | (4) | (5) | (6) |
| --- | --- | --- | --- | --- | --- | --- |
|  | BRCS-O | BRCS-COVID-19 | Domestic Work | Ag/Paid Work | Domestic or Ag/Paid Work | GCBS |
| Age | 0.061*** | 0.023** | 0.036*** | 0.007 | 0.035*** | 0.011 |
|  | (0.010) | (0.010) | (0.009) | (0.006) | (0.008) | (0.009) |
| Above median on asset index | 0.048 | 0.101** | -0.073* | -0.031 | -0.079** | -0.122*** |
|  | (0.048) | (0.048) | (0.044) | (0.025) | (0.040) | (0.044) |
| Ever Married | 0.174** | -0.045 | 0.425*** | -0.001 | 0.353*** | 0.145* |
|  | (0.078) | (0.079) | (0.075) | (0.063) | (0.071) | (0.077) |
| Disability status | -0.098 | -0.110* | -0.103 | -0.051** | -0.116** | 0.147** |
|  | (0.070) | (0.065) | (0.064) | (0.023) | (0.056) | (0.062) |
| Enrolled in school March 2020 | 0.354*** | 0.105 | -0.199*** | -0.096* | -0.223*** | -0.047 |
|  | (0.073) | (0.073) | (0.063) | (0.057) | (0.063) | (0.066) |
| Eth: South Gondor (rural) | 0.108 | -0.111 | 0.180* | 0.401*** | 0.368*** | -0.002 |
|  | (0.113) | (0.106) | (0.095) | (0.060) | (0.088) | (0.086) |
| Eth: East Haraghe (rural) | 0.211* | 0.336*** | 0.294*** | -0.042 | 0.183** | 0.317*** |
|  | (0.120) | (0.110) | (0.092) | (0.044) | (0.080) | (0.100) |
| Eth: Debre Tabor (urban) | 0.108 | 0.117 | 0.016 | -0.110** | -0.074 | 0.175** |
|  | (0.094) | (0.083) | (0.103) | (0.049) | (0.085) | (0.086) |
| Eth: Dire Dawa (urban) | 0.182* | 0.399*** | -0.056 | -0.046 | -0.089 | 0.362*** |
|  | (0.110) | (0.129) | (0.102) | (0.075) | (0.095) | (0.100) |
| Jord: Syrian refugee living in host community | 0.214** | 0.163** | 0.100 | -0.075** | 0.040 | 0.187** |
|  | (0.083) | (0.083) | (0.088) | (0.031) | (0.077) | (0.082) |
| Jord: Syrian refugee living in camp | 0.245** | 0.299*** | 0.102 | -0.052 | 0.058 | 0.082 |
|  | (0.096) | (0.096) | (0.097) | (0.034) | (0.086) | (0.089) |
| Jord: Syrian refugee living in ITS | 0.023 | 0.308** | 0.141 | 0.344** | 0.343*** | 0.273* |
|  | (0.131) | (0.125) | (0.125) | (0.138) | (0.129) | (0.142) |
| Jord: Palestinian refugee | -0.034 | -0.029 | 0.021 | 0.048 | 0.051 | 0.271** |
|  | (0.114) | (0.117) | (0.117) | (0.080) | (0.109) | (0.118) |
| Jord: Other nationality | 0.192 | 0.260 | -0.419** | -0.116*** | -0.420*** | -0.066 |
|  | (0.215) | (0.245) | (0.182) | (0.042) | (0.157) | (0.217) |
| Pal: Gaza | 0.112 | 0.458*** | 0.459*** | -0.011 | 0.393*** | 0.422*** |
|  | (0.116) | (0.115) | (0.131) | (0.035) | (0.114) | (0.122) |
| Pal: West Bank | 0.460*** | 0.468*** | -0.024 | 0.033 | 0.003 | -0.364*** |
|  | (0.091) | (0.095) | (0.097) | (0.030) | (0.085) | (0.084) |
| Pal: Lives in a refugee camp | -0.320** | -0.601*** | -0.171 | 0.039 | -0.123 | 0.002 |
|  | (0.135) | (0.122) | (0.108) | (0.037) | (0.096) | (0.103) |
| Pal: Refugee not living in a camp | 0.080 | -0.223* | -0.178 | 0.023 | -0.139 | 0.087 |
|  | (0.124) | (0.132) | (0.160) | (0.020) | (0.137) | (0.146) |
| Number of observations | 2,345 | 2,348 | 2,964 | 2,984 | 2,964 | 2,864 |

*Notes.*  Each panel of the table presents separate specification. Each column is a separate regression of the outcome specified in the header on the covariates listed in the rows. Eth=Ethiopia, Jord=Jordan, Pal=Palestine. See Table 1 for notes on covariates. Standard errors are clustered at the *subkebele* level in Ethiopia to account for sampling design, and at the individual level in Jordan and Palestine and presented in parentheses. *** p<.01, ** p<.05, * p<.10

T**able B3. Covariate coefficients, boys only (Ethiopia, Jordan, Palestine)**

|  | (1) | (2) | (3) | (4) | (5) |
| --- | --- | --- | --- | --- | --- |
|  | BRCS-O | BRCS-COVID-19 | Domestic Work | Ag/Paid Work | Domestic or Ag/Paid Work |
| Age | 0.043*** | -0.000 | 0.005 | 0.061*** | 0.041*** |
|  | (0.010) | (0.010) | (0.008) | (0.011) | (0.010) |
| Above median on asset index | 0.014 | 0.100** | -0.038 | 0.010 | -0.026 |
|  | (0.047) | (0.048) | (0.041) | (0.055) | (0.049) |
| Ever Married | 0.241 | 0.381** | 0.112 | 0.698** | 0.541** |
|  | (0.176) | (0.162) | (0.208) | (0.319) | (0.240) |
| Disability status | -0.147** | -0.085 | 0.033 | -0.168** | -0.074 |
|  | (0.067) | (0.065) | (0.053) | (0.069) | (0.063) |
| Enrolled in school March 2020 | 0.146** | 0.134** | 0.152*** | -0.427*** | -0.137** |
|  | (0.061) | (0.063) | (0.049) | (0.083) | (0.062) |
| Eth: South Gondor (rural) | -0.378*** | 0.101 | 0.066 | 0.829*** | 0.596*** |
|  | (0.126) | (0.140) | (0.095) | (0.135) | (0.116) |
| Eth: East Haraghe (rural) | 0.204 | 0.327** | -0.066 | 0.217 | 0.117 |
|  | (0.133) | (0.154) | (0.099) | (0.133) | (0.118) |
| Eth: Debre Tabor (urban) | -0.447*** | 0.019 | 0.181* | -0.283** | -0.001 |
|  | (0.132) | (0.147) | (0.098) | (0.128) | (0.119) |
| Eth: Dire Dawa (urban) | 0.128 | 0.468*** | -0.129 | -0.457*** | -0.342*** |
|  | (0.112) | (0.127) | (0.094) | (0.137) | (0.114) |
| Jord: Syrian refugee living in host community | -0.002 | 0.281*** | 0.091 | 0.124 | 0.147 |
|  | (0.105) | (0.109) | (0.083) | (0.126) | (0.104) |
| Jord: Syrian refugee living in camp | -0.024 | 0.457*** | 0.095 | -0.058 | 0.047 |
|  | (0.115) | (0.116) | (0.087) | (0.132) | (0.110) |
| Jord: Syrian refugee living in ITS | -0.189 | 0.512*** | 0.335*** | -0.160 | 0.181 |
|  | (0.147) | (0.146) | (0.122) | (0.160) | (0.146) |
| Jord: Palestinian refugee | -0.372** | 0.062 | 0.320** | 0.100 | 0.331** |
|  | (0.147) | (0.159) | (0.139) | (0.168) | (0.158) |
| Jord: Other nationality | -0.010 | 0.058 | 0.059 | -0.351** | -0.160 |
|  | (0.204) | (0.213) | (0.205) | (0.171) | (0.203) |
| Pal: Gaza | -0.134 | 0.462*** | 0.403*** | 0.123 | 0.416*** |
|  | (0.139) | (0.132) | (0.113) | (0.160) | (0.137) |
| Pal: West Bank | 0.160 | 0.488*** | -0.157** | 0.307* | 0.048 |
|  | (0.111) | (0.114) | (0.079) | (0.162) | (0.119) |
| Pal: Lives in a refugee camp | -0.427*** | -0.826*** | -0.260*** | -0.406*** | -0.475*** |
|  | (0.139) | (0.122) | (0.066) | (0.114) | (0.090) |
| Pal: Refugee not living in a camp | -0.009 | -0.170 | -0.078 | -0.229 | -0.208 |
|  | (0.121) | (0.122) | (0.111) | (0.162) | (0.136) |
| Number of observations | 2,232 | 2,233 | 2,736 | 2,750 | 2,736 |

*Notes.*  Each panel of the table presents separate specification. Each column is a separate regression of the outcome specified in the header on the covariates listed in the rows. Eth=Ethiopia, Jord=Jordan, Pal=Palestine. See Table 1 for notes on covariates. Standard errors are clustered at the *subkebele* level in Ethiopia to account for sampling design, and at the individual level in Jordan and Palestine and presented in parentheses. *** p<.01, ** p<.05, * p<.10

T**able B4. Multivariate regression results for the Ethiopia sample – COVID-19 vulnerability, social protection, and interaction (boys and girls)**

|  | (1) | (2) | (3) | (4) | (5) |
| --- | --- | --- | --- | --- | --- |
|  | BRCS-O | BRCS-COVID-19 | Domestic Work | Ag/Paid Work | Domestic or Ag/Paid Work |
| COVID-19 vulnerability | -0.030 | 0.002 | 0.032 | 0.053** | 0.057** |
|  | (0.030) | (0.033) | (0.023) | (0.023) | (0.023) |
| Received social protection | -0.012 | 0.147* | -0.009 | 0.071 | 0.039 |
|  | (0.107) | (0.088) | (0.057) | (0.066) | (0.060) |
| Vulnerable x social protection | 0.011 | -0.003 | -0.025 | -0.080 | -0.069 |
|  | (0.109) | (0.098) | (0.064) | (0.080) | (0.079) |
| Female | 0.013 | -0.059 | 0.634*** | -0.414*** | 0.209*** |
|  | (0.063) | (0.063) | (0.046) | (0.045) | (0.049) |
| Age | 0.036*** | -0.016 | 0.012 | 0.056*** | 0.044*** |
|  | (0.014) | (0.012) | (0.009) | (0.010) | (0.009) |
| Above median on asset index | -0.065 | 0.114 | -0.040 | -0.010 | -0.036 |
|  | (0.091) | (0.089) | (0.060) | (0.055) | (0.062) |
| Ever Married | -0.007 | 0.149 | 0.173* | -0.117 | 0.054 |
|  | (0.143) | (0.137) | (0.101) | (0.119) | (0.102) |
| Disability status | -0.316** | -0.328** | -0.163 | -0.185** | -0.238** |
|  | (0.143) | (0.143) | (0.107) | (0.081) | (0.102) |
| Enrolled in school March 2020 | 0.111 | -0.083 | -0.067 | -0.297*** | -0.238** |
|  | (0.121) | (0.115) | (0.085) | (0.111) | (0.094) |
| Eth: East Haraghe (rural) | 0.264*** | 0.330*** | -0.005 | -0.486*** | -0.313*** |
|  | (0.087) | (0.096) | (0.054) | (0.052) | (0.054) |
| Eth: Debre Tabor (urban) | 0.022 | 0.166* | -0.006 | -0.757*** | -0.485*** |
|  | (0.104) | (0.093) | (0.069) | (0.067) | (0.067) |
| Eth: Dire Dawa (urban) | 0.337*** | 0.503*** | -0.208*** | -0.843*** | -0.690*** |
|  | (0.108) | (0.105) | (0.072) | (0.069) | (0.067) |
| Constant | -0.774*** | 0.055 | -0.406** | 0.128 | -0.221 |
|  | (0.252) | (0.235) | (0.165) | (0.166) | (0.166) |
| Number of observations | 1,131 | 1,132 | 2,296 | 2,296 | 2,296 |

*Notes.*  Each panel of the table presents separate specification. Each column is a separate regression of the outcome specified in the header on the covariates listed in the rows. Eth=Ethiopia, Jord=Jordan, Pal=Palestine. See Table 1 for notes on covariates. Standard errors are clustered at the *subkebele* level in Ethiopia to account for sampling design, and at the individual level in Jordan and Palestine and presented in parentheses. In the Ethiopia-specific estimates, adolescents living in South Gondor are the reference group. *** p<.01, ** p<.05, * p<.10

T**able B5. Multivariate regression results for the Ethiopia sample – COVID-19 vulnerability, social protection, and interaction, girls only**

|  | (1) | (2) | (3) | (4) | (5) | (6) |
| --- | --- | --- | --- | --- | --- | --- |
|  | BRCS-O | BRCS-COVID-19 | Domestic Work | Ag/Paid Work | Domestic or Ag/Paid Work | GCBS |
| COVID-19 vulnerability | -0.052 | 0.035 | 0.015 | 0.048 | 0.042 | 0.077** |
|  | (0.043) | (0.046) | (0.030) | (0.030) | (0.030) | (0.035) |
| Received social protection | -0.090 | 0.170 | -0.058 | 0.038 | -0.019 | -0.013 |
|  | (0.153) | (0.125) | (0.077) | (0.065) | (0.072) | (0.082) |
| Vulnerable x social protection | 0.073 | -0.060 | 0.055 | -0.014 | 0.032 | 0.059 |
|  | (0.158) | (0.122) | (0.084) | (0.075) | (0.075) | (0.093) |
| Age | 0.038** | -0.006 | 0.004 | 0.034*** | 0.025** | 0.025* |
|  | (0.018) | (0.017) | (0.012) | (0.013) | (0.012) | (0.015) |
| Above median on asset index | -0.042 | 0.264** | -0.085 | -0.136** | -0.150* | 0.127 |
|  | (0.116) | (0.108) | (0.077) | (0.062) | (0.078) | (0.083) |
| Ever Married | -0.042 | 0.096 | 0.184 | -0.105 | 0.070 | 0.121 |
|  | (0.160) | (0.164) | (0.114) | (0.141) | (0.121) | (0.124) |
| Disability status | -0.224 | -0.359** | -0.256* | -0.201** | -0.318*** | -0.112 |
|  | (0.176) | (0.164) | (0.132) | (0.089) | (0.121) | (0.124) |
| Enrolled in school March 2020 | 0.099 | -0.076 | -0.169 | -0.338** | -0.340*** | 0.004 |
|  | (0.161) | (0.149) | (0.111) | (0.145) | (0.128) | (0.124) |
| Eth: East Haraghe (rural) | 0.055 | 0.436*** | 0.071 | -0.457*** | -0.237*** | 0.367*** |
|  | (0.122) | (0.113) | (0.074) | (0.061) | (0.073) | (0.082) |
| Eth: Debre Tabor (urban) | 0.047 | 0.233** | -0.088 | -0.458*** | -0.356*** | 0.070 |
|  | (0.142) | (0.112) | (0.104) | (0.075) | (0.092) | (0.098) |
| Eth: Dire Dawa (urban) | 0.128 | 0.498*** | -0.165 | -0.431*** | -0.396*** | 0.233** |
|  | (0.153) | (0.146) | (0.100) | (0.089) | (0.092) | (0.111) |
| Constant | -0.701** | -0.291 | 0.462** | -0.009 | 0.337 | -0.632** |
|  | (0.306) | (0.298) | (0.229) | (0.198) | (0.227) | (0.259) |
| Number of observations | 617 | 618 | 1,259 | 1,259 | 1,259 | 1,260 |

*Notes.*  Each panel of the table presents separate specification. Each column is a separate regression of the outcome specified in the header on the covariates listed in the rows. Eth=Ethiopia, Jord=Jordan, Pal=Palestine. See Table 1 for notes on covariates. Standard errors are clustered at the *subkebele* level in Ethiopia to account for sampling design, and at the individual level in Jordan and Palestine and presented in parentheses. In the Ethiopia-specific estimates, adolescents living in South Gondor are the reference group. *** p<.01, ** p<.05, * p<.10

T**able B6. Multivariate regression results for the Ethiopia sample – COVID-19 vulnerability, social protection, and interaction, boys only**

|  | (1) | (2) | (3) | (4) | (5) |
| --- | --- | --- | --- | --- | --- |
|  | BRCS-O | BRCS-COVID-19 | Domestic Work | Ag/Paid Work | Domestic or Ag/Paid Work |
| COVID-19 vulnerability | -0.003 | -0.036 | 0.054* | 0.052 | 0.073** |
|  | (0.048) | (0.053) | (0.031) | (0.036) | (0.035) |
| Received social protection | 0.026 | 0.110 | 0.033 | 0.074 | 0.072 |
|  | (0.123) | (0.143) | (0.083) | (0.103) | (0.096) |
| Vulnerable x social protection | -0.038 | 0.075 | -0.104 | -0.103 | -0.143 |
|  | (0.133) | (0.148) | (0.082) | (0.121) | (0.116) |
| Age | 0.027 | -0.024 | 0.028** | 0.085*** | 0.075*** |
|  | (0.020) | (0.021) | (0.014) | (0.015) | (0.015) |
| Above median on asset index | -0.148 | -0.066 | 0.048 | 0.175* | 0.147 |
|  | (0.130) | (0.138) | (0.099) | (0.101) | (0.110) |
| Ever Married | -0.044 | 0.466 | -0.374 | 0.559 | 0.078 |
|  | (0.406) | (0.289) | (0.233) | (0.383) | (0.262) |
| Disability status | -0.463* | -0.329 | -0.042 | -0.190 | -0.152 |
|  | (0.252) | (0.259) | (0.157) | (0.150) | (0.166) |
| Enrolled in school March 2020 | 0.019 | -0.098 | 0.161 | -0.219 | -0.019 |
|  | (0.172) | (0.182) | (0.138) | (0.199) | (0.124) |
| Eth: East Haraghe (rural) | 0.555*** | 0.208 | -0.128 | -0.577*** | -0.461*** |
|  | (0.122) | (0.148) | (0.086) | (0.090) | (0.092) |
| Eth: Debre Tabor (urban) | 0.064 | 0.065 | 0.046 | -1.185*** | -0.718*** |
|  | (0.146) | (0.160) | (0.113) | (0.115) | (0.129) |
| Eth: Dire Dawa (urban) | 0.616*** | 0.501*** | -0.293*** | -1.381*** | -1.094*** |
|  | (0.143) | (0.160) | (0.105) | (0.119) | (0.119) |
| Constant | -0.628* | 0.354 | -0.892*** | -0.258 | -0.827*** |
|  | (0.367) | (0.416) | (0.263) | (0.306) | (0.271) |
| Number of observations | 514 | 514 | 1,037 | 1,037 | 1,037 |

*Notes.*  Each panel of the table presents separate specification. Each column is a separate regression of the outcome specified in the header on the covariates listed in the rows. Eth=Ethiopia, Jord=Jordan, Pal=Palestine. See Table 1 for notes on covariates. Standard errors are clustered at the *subkebele* level in Ethiopia to account for sampling design, and at the individual level in Jordan and Palestine and presented in parentheses. In the Ethiopia-specific estimates, adolescents living in South Gondor are the reference group. *** p<.01, ** p<.05, * p<.10

T**able B7. Multivariate regression results for the Jordan sample – COVID-19 vulnerability, social protection, and interaction (boys and girls)**

|  | (1) | (2) | (3) | (4) | (5) |
| --- | --- | --- | --- | --- | --- |
|  | BRCS-O | BRCS-COVID-19 | Domestic Work | Ag/Paid Work | Domestic or Ag/Paid Work |
| COVID-19 vulnerability | -0.116** | -0.067 | 0.046 | -0.054 | 0.001 |
|  | (0.046) | (0.050) | (0.047) | (0.039) | (0.045) |
| Received social protection | 0.200** | 0.051 | -0.162* | -0.006 | -0.132 |
|  | (0.082) | (0.086) | (0.090) | (0.074) | (0.085) |
| Vulnerable x social protection | 0.103** | 0.069 | 0.054 | 0.048 | 0.082 |
|  | (0.051) | (0.055) | (0.052) | (0.046) | (0.051) |
| Female | -0.134*** | -0.082** | 0.498*** | -0.494*** | 0.126*** |
|  | (0.041) | (0.041) | (0.038) | (0.040) | (0.039) |
| Age | 0.067*** | 0.009 | 0.022** | 0.016* | 0.028*** |
|  | (0.009) | (0.010) | (0.009) | (0.009) | (0.009) |
| Above median on asset index | 0.034 | 0.042 | -0.048 | -0.024 | -0.056 |
|  | (0.043) | (0.044) | (0.042) | (0.042) | (0.042) |
| Ever Married | 0.193** | -0.061 | 0.777*** | -0.131* | 0.586*** |
|  | (0.087) | (0.091) | (0.107) | (0.078) | (0.098) |
| Disability status | 0.006 | -0.012 | 0.043 | -0.043 | 0.013 |
|  | (0.058) | (0.056) | (0.054) | (0.055) | (0.057) |
| Enrolled in school March 2020 | 0.276*** | 0.050 | 0.035 | -0.229*** | -0.107* |
|  | (0.057) | (0.059) | (0.053) | (0.063) | (0.058) |
| Jord: Syrian refugee living in host community | 0.029 | 0.174* | 0.199* | 0.086 | 0.214** |
|  | (0.100) | (0.101) | (0.107) | (0.084) | (0.101) |
| Jord: Syrian refugee living in camp | 0.045 | 0.345*** | 0.170 | 0.009 | 0.147 |
|  | (0.107) | (0.107) | (0.111) | (0.088) | (0.106) |
| Jord: Syrian refugee living in ITS | -0.123 | 0.350*** | 0.256** | 0.179 | 0.322** |
|  | (0.125) | (0.122) | (0.127) | (0.119) | (0.126) |
| Jord: Palestinian refugee | -0.185* | -0.013 | 0.200** | 0.080 | 0.217** |
|  | (0.095) | (0.100) | (0.097) | (0.086) | (0.097) |
| Jord: Other nationality | 0.041 | 0.090 | -0.060 | -0.262*** | -0.202 |
|  | (0.162) | (0.167) | (0.149) | (0.094) | (0.133) |
| Constant | -1.362*** | -0.382** | -0.703*** | 0.134 | -0.524*** |
|  | (0.185) | (0.194) | (0.174) | (0.159) | (0.175) |
| Number of observations | 2,533 | 2,533 | 2,488 | 2,522 | 2,488 |

*Notes.*  Each panel of the table presents separate specification. Each column is a separate regression of the outcome specified in the header on the covariates listed in the rows. Eth=Ethiopia, Jord=Jordan, Pal=Palestine. See Table 1 for notes on covariates. Standard errors are clustered at the *subkebele* level in Ethiopia to account for sampling design, and at the individual level in Jordan and Palestine and presented in parentheses. In the Jordan-specific estimates, Jordanian adolescents are the reference group. *** p<.01, ** p<.05, * p<.10

T**able B8. Multivariate regression results for the Jordan sample – COVID-19 vulnerability, social protection, and interaction, girls only**

|  | (1) | (2) | (3) | (4) | (5) | (6) |
| --- | --- | --- | --- | --- | --- | --- |
|  | BRCS-O | BRCS-COVID-19 | Domestic Work | Ag/Paid Work | Domestic or Ag/Paid Work | GCBS |
| COVID-19 vulnerability | -0.111* | -0.137** | 0.063 | 0.036* | 0.066 | 0.079 |
|  | (0.059) | (0.067) | (0.060) | (0.021) | (0.050) | (0.062) |
| Received social protection | 0.343*** | 0.090 | -0.095 | -0.030 | -0.079 | -0.010 |
|  | (0.115) | (0.121) | (0.123) | (0.062) | (0.108) | (0.124) |
| Vulnerable x social protection | 0.086 | 0.102 | 0.071 | -0.003 | 0.068 | 0.017 |
|  | (0.068) | (0.076) | (0.069) | (0.026) | (0.059) | (0.070) |
| Age | 0.077*** | 0.022 | 0.055*** | -0.011 | 0.040*** | -0.002 |
|  | (0.013) | (0.014) | (0.014) | (0.008) | (0.013) | (0.014) |
| Above median on asset index | 0.027 | -0.033 | -0.034 | 0.032 | -0.009 | -0.169*** |
|  | (0.061) | (0.062) | (0.063) | (0.029) | (0.055) | (0.062) |
| Ever Married | 0.246** | -0.124 | 0.597*** | -0.035 | 0.495*** | 0.111 |
|  | (0.107) | (0.108) | (0.119) | (0.050) | (0.105) | (0.118) |
| Disability status | 0.037 | -0.002 | 0.055 | -0.042** | 0.022 | 0.319*** |
|  | (0.083) | (0.077) | (0.081) | (0.021) | (0.071) | (0.085) |
| Enrolled in school March 2020 | 0.400*** | 0.059 | -0.133 | -0.052 | -0.140* | -0.089 |
|  | (0.086) | (0.088) | (0.084) | (0.054) | (0.077) | (0.090) |
| Jord: Syrian refugee living in host community | -0.038 | 0.114 | 0.146 | -0.020 | 0.099 | 0.127 |
|  | (0.132) | (0.133) | (0.145) | (0.067) | (0.127) | (0.149) |
| Jord: Syrian refugee living in camp | 0.020 | 0.293** | 0.102 | -0.030 | 0.058 | -0.006 |
|  | (0.142) | (0.141) | (0.151) | (0.066) | (0.133) | (0.154) |
| Jord: Syrian refugee living in ITS | -0.187 | 0.258 | 0.112 | 0.401*** | 0.334** | 0.120 |
|  | (0.171) | (0.165) | (0.177) | (0.143) | (0.166) | (0.193) |
| Jord: Palestinian refugee | -0.120 | -0.065 | 0.073 | 0.068 | 0.100 | 0.235* |
|  | (0.120) | (0.123) | (0.129) | (0.088) | (0.120) | (0.125) |
| Jord: Other nationality | 0.047 | 0.150 | -0.428** | -0.050 | -0.396** | -0.153 |
|  | (0.225) | (0.260) | (0.189) | (0.046) | (0.161) | (0.224) |
| Constant | -1.827*** | -0.617** | -0.571** | -0.048 | -0.526** | 0.032 |
|  | (0.250) | (0.269) | (0.263) | (0.136) | (0.237) | (0.267) |
| Number of observations | 1,282 | 1,283 | 1,257 | 1,277 | 1,257 | 1,199 |

*Notes.*  Each panel of the table presents separate specification. Each column is a separate regression of the outcome specified in the header on the covariates listed in the rows. Eth=Ethiopia, Jord=Jordan, Pal=Palestine. See Table 1 for notes on covariates. Standard errors are clustered at the *subkebele* level in Ethiopia to account for sampling design, and at the individual level in Jordan and Palestine and presented in parentheses. In the Jordan-specific estimates, Jordanian adolescents are the reference group. *** p<.01, ** p<.05, * p<.10

T**able B9. Multivariate regression results for the Jordan sample – COVID-19 vulnerability, social protection, and interaction, boys only**

|  | (1) | (2) | (3) | (4) | (5) |
| --- | --- | --- | --- | --- | --- |
|  | BRCS-O | BRCS-COVID-19 | Domestic Work | Ag/Paid Work | Domestic or Ag/Paid Work |
| COVID-19 vulnerability | -0.140* | 0.015 | 0.008 | -0.153* | -0.085 |
|  | (0.075) | (0.075) | (0.075) | (0.087) | (0.079) |
| Received social protection | -0.002 | 0.017 | -0.227* | -0.078 | -0.241* |
|  | (0.112) | (0.125) | (0.131) | (0.149) | (0.137) |
| Vulnerable x social protection | 0.138* | 0.026 | 0.053 | 0.113 | 0.115 |
|  | (0.080) | (0.080) | (0.080) | (0.095) | (0.086) |
| Age | 0.053*** | -0.006 | -0.008 | 0.037** | 0.016 |
|  | (0.014) | (0.014) | (0.012) | (0.016) | (0.014) |
| Above median on asset index | 0.041 | 0.120* | -0.070 | -0.074 | -0.106 |
|  | (0.059) | (0.062) | (0.055) | (0.077) | (0.065) |
| Ever Married | 0.346** | 0.367* | 0.444 | 0.859* | 0.898*** |
|  | (0.166) | (0.192) | (0.307) | (0.453) | (0.343) |
| Disability status | -0.033 | -0.040 | 0.039 | -0.049 | 0.008 |
|  | (0.080) | (0.081) | (0.071) | (0.108) | (0.089) |
| Enrolled in school March 2020 | 0.150* | 0.019 | 0.123* | -0.352*** | -0.106 |
|  | (0.078) | (0.081) | (0.067) | (0.105) | (0.083) |
| Jord: Syrian refugee living in host community | 0.117 | 0.249 | 0.281* | 0.315* | 0.424** |
|  | (0.162) | (0.162) | (0.154) | (0.181) | (0.166) |
| Jord: Syrian refugee living in camp | 0.095 | 0.412** | 0.280* | 0.155 | 0.334* |
|  | (0.171) | (0.169) | (0.159) | (0.191) | (0.174) |
| Jord: Syrian refugee living in ITS | -0.049 | 0.419** | 0.436** | 0.081 | 0.417** |
|  | (0.195) | (0.188) | (0.178) | (0.205) | (0.194) |
| Jord: Palestinian refugee | -0.313** | 0.051 | 0.369** | 0.149 | 0.403** |
|  | (0.157) | (0.168) | (0.148) | (0.170) | (0.160) |
| Jord: Other nationality | 0.079 | 0.059 | 0.174 | -0.209 | 0.025 |
|  | (0.238) | (0.227) | (0.219) | (0.181) | (0.209) |
| Constant | -0.949*** | -0.199 | -0.338 | -0.187 | -0.401 |
|  | (0.272) | (0.279) | (0.218) | (0.291) | (0.252) |
| Number of observations | 1,251 | 1,250 | 1,231 | 1,245 | 1,231 |

*Notes.*  Each panel of the table presents separate specification. Each column is a separate regression of the outcome specified in the header on the covariates listed in the rows. Eth=Ethiopia, Jord=Jordan, Pal=Palestine. See Table 1 for notes on covariates. Standard errors are clustered at the *subkebele* level in Ethiopia to account for sampling design, and at the individual level in Jordan and Palestine and presented in parentheses. In the Jordan-specific estimates, Jordanian adolescents are the reference group. *** p<.01, ** p<.05, * p<.10

T**able B10. Multivariate regression results for the Palestine sample – COVID-19 vulnerability, social protection, and interaction (boys and girls)**

|  | (1) | (2) | (3) | (4) | (5) |
| --- | --- | --- | --- | --- | --- |
|  | BRCS-O | BRCS-COVID-19 | Domestic Work | Ag/Paid Work | Domestic or Ag/Paid Work |
| COVID-19 vulnerability | -0.118** | -0.160*** | -0.045 | -0.030 | -0.058 |
|  | (0.053) | (0.051) | (0.044) | (0.044) | (0.044) |
| Received social protection | -0.180* | -0.003 | -0.007 | 0.085 | 0.045 |
|  | (0.106) | (0.097) | (0.097) | (0.070) | (0.090) |
| Vulnerable x social protection | 0.110 | 0.027 | 0.213*** | 0.012 | 0.192*** |
|  | (0.067) | (0.064) | (0.066) | (0.053) | (0.064) |
| Female | -0.035 | -0.004 | 0.537*** | -0.274*** | 0.301*** |
|  | (0.072) | (0.065) | (0.058) | (0.051) | (0.058) |
| Age | 0.019 | 0.036** | 0.052*** | 0.055*** | 0.078*** |
|  | (0.016) | (0.015) | (0.015) | (0.018) | (0.016) |
| Above median on asset index | -0.041 | 0.064 | 0.054 | 0.044 | 0.073 |
|  | (0.078) | (0.076) | (0.062) | (0.074) | (0.067) |
| Ever Married | 0.272** | 0.189 | 1.030*** | -0.787*** | 0.418** |
|  | (0.127) | (0.126) | (0.163) | (0.154) | (0.164) |
| Disability status | -0.269*** | -0.082 | -0.120 | -0.273*** | -0.270*** |
|  | (0.104) | (0.089) | (0.079) | (0.064) | (0.077) |
| Enrolled in school March 2020 | 0.257** | 0.416*** | 0.125* | -0.802*** | -0.377*** |
|  | (0.111) | (0.102) | (0.075) | (0.169) | (0.116) |
| Pal: Gaza | -0.170* | 0.087 | 0.518*** | -0.094 | 0.393*** |
|  | (0.102) | (0.093) | (0.098) | (0.075) | (0.091) |
| Pal: Lives in a refugee camp | -0.321*** | -0.657*** | -0.186*** | -0.220*** | -0.295*** |
|  | (0.095) | (0.083) | (0.062) | (0.068) | (0.068) |
| Pal: Refugee not living in a camp | 0.072 | -0.182** | -0.090 | -0.149 | -0.168* |
|  | (0.088) | (0.085) | (0.095) | (0.091) | (0.097) |
| Constant | -0.181 | -0.707*** | -1.500*** | 0.142 | -1.217*** |
|  | (0.278) | (0.268) | (0.257) | (0.309) | (0.279) |
| Number of observations | 913 | 916 | 916 | 916 | 916 |

*Notes.*  Each panel of the table presents separate specification. Each column is a separate regression of the outcome specified in the header on the covariates listed in the rows. Eth=Ethiopia, Jord=Jordan, Pal=Palestine. See Table 1 for notes on covariates. Standard errors are clustered at the *subkebele* level in Ethiopia to account for sampling design, and at the individual level in Jordan and Palestine and presented in parentheses. In the Palestine-specific estimates, adolescents living in the West Bank and non-refugees are the reference group. *** p<.01, ** p<.05, * p<.10

T**able B11. Multivariate regression results for the Palestine sample – COVID-19 vulnerability, social protection, and interaction, girls only**

|  | (1) | (2) | (3) | (4) | (5) | (6) |
| --- | --- | --- | --- | --- | --- | --- |
|  | BRCS-O | BRCS-COVID-19 | Domestic Work | Ag/Paid Work | Domestic or Ag/Paid Work | GCBS |
| COVID-19 vulnerability | -0.063 | -0.098 | -0.085 | 0.021 | -0.061 | 0.262*** |
|  | (0.073) | (0.070) | (0.065) | (0.016) | (0.057) | (0.084) |
| Received social protection | -0.195 | 0.004 | 0.098 | -0.024 | 0.071 | -0.216 |
|  | (0.154) | (0.153) | (0.149) | (0.024) | (0.131) | (0.135) |
| Vulnerable x social protection | 0.127 | -0.014 | 0.191* | -0.015 | 0.157* | 0.141 |
|  | (0.092) | (0.090) | (0.101) | (0.014) | (0.088) | (0.098) |
| Age | 0.025 | 0.049** | 0.099*** | 0.012 | 0.094*** | 0.043** |
|  | (0.024) | (0.023) | (0.025) | (0.009) | (0.023) | (0.020) |
| Above median on asset index | 0.054 | 0.158 | 0.035 | -0.012 | 0.024 | -0.120 |
|  | (0.117) | (0.117) | (0.109) | (0.030) | (0.097) | (0.097) |
| Ever Married | 0.598** | 0.247 | 0.643*** | 0.076 | 0.605*** | -0.206 |
|  | (0.243) | (0.226) | (0.216) | (0.096) | (0.215) | (0.218) |
| Disability status | -0.318** | -0.159 | -0.309** | -0.007 | -0.272** | -0.222* |
|  | (0.160) | (0.139) | (0.139) | (0.020) | (0.122) | (0.124) |
| Enrolled in school March 2020 | 0.695** | 0.537** | -0.198 | 0.128 | -0.094 | -0.038 |
|  | (0.269) | (0.251) | (0.205) | (0.092) | (0.202) | (0.210) |
| Pal: Gaza | -0.220 | 0.098 | 0.496*** | -0.040 | 0.406*** | 0.674*** |
|  | (0.159) | (0.152) | (0.152) | (0.027) | (0.133) | (0.128) |
| Pal: Lives in a refugee camp | -0.242* | -0.522*** | -0.186* | 0.063 | -0.124 | -0.005 |
|  | (0.132) | (0.119) | (0.105) | (0.044) | (0.097) | (0.103) |
| Pal: Refugee not living in a camp | 0.145 | -0.185 | -0.183 | 0.040* | -0.134 | 0.126 |
|  | (0.121) | (0.128) | (0.156) | (0.024) | (0.136) | (0.139) |
| Constant | -0.760* | -1.080** | -1.330*** | -0.508*** | -1.463*** | -0.845** |
|  | (0.458) | (0.448) | (0.482) | (0.183) | (0.444) | (0.369) |
| Number of observations | 446 | 447 | 448 | 448 | 448 | 405 |

*Notes.*  Each panel of the table presents separate specification. Each column is a separate regression of the outcome specified in the header on the covariates listed in the rows. Eth=Ethiopia, Jord=Jordan, Pal=Palestine. See Table 1 for notes on covariates. Standard errors are clustered at the *subkebele* level in Ethiopia to account for sampling design, and at the individual level in Jordan and Palestine and presented in parentheses. In the Palestine-specific estimates, adolescents living in the West Bank and non-refugees are the reference group. *** p<.01, ** p<.05, * p<.10

T**able B12. Multivariate regression results for the Palestine sample – COVID-19 vulnerability, social protection, and interaction, boys only**

|  | (1) | (2) | (3) | (4) | (5) |
| --- | --- | --- | --- | --- | --- |
|  | BRCS-O | BRCS-COVID-19 | Domestic Work | Ag/Paid Work | Domestic or Ag/Paid Work |
| COVID-19 vulnerability | -0.182** | -0.227*** | 0.010 | -0.091 | -0.046 |
|  | (0.079) | (0.074) | (0.059) | (0.090) | (0.070) |
| Received social protection | -0.186 | -0.028 | -0.171 | 0.232* | -0.008 |
|  | (0.146) | (0.121) | (0.119) | (0.138) | (0.124) |
| Vulnerable x social protection | 0.097 | 0.070 | 0.208** | 0.065 | 0.220** |
|  | (0.100) | (0.094) | (0.083) | (0.106) | (0.094) |
| Age | 0.011 | 0.029 | 0.012 | 0.093*** | 0.067*** |
|  | (0.021) | (0.021) | (0.016) | (0.033) | (0.023) |
| Above median on asset index | -0.125 | -0.028 | 0.053 | 0.133 | 0.127 |
|  | (0.103) | (0.097) | (0.066) | (0.133) | (0.094) |
| Ever Married | -- | -- | -- | -- | -- |
|  | -- | -- | -- | -- | -- |
| Disability status | -0.181 | 0.009 | -0.006 | -0.410*** | -0.254** |
|  | (0.134) | (0.116) | (0.090) | (0.112) | (0.100) |
| Enrolled in school March 2020 | 0.115 | 0.384*** | 0.166** | -0.988*** | -0.455*** |
|  | (0.119) | (0.111) | (0.075) | (0.214) | (0.143) |
| Pal: Gaza | -0.103 | 0.089 | 0.609*** | -0.225 | 0.393*** |
|  | (0.128) | (0.114) | (0.118) | (0.151) | (0.127) |
| Pal: Lives in a refugee camp | -0.357*** | -0.772*** | -0.208*** | -0.423*** | -0.436*** |
|  | (0.137) | (0.119) | (0.069) | (0.123) | (0.096) |
| Pal: Refugee not living in a camp | 0.016 | -0.174 | -0.017 | -0.287* | -0.188 |
|  | (0.126) | (0.115) | (0.111) | (0.173) | (0.139) |
| Constant | 0.059 | -0.505 | -0.929*** | -0.236 | -0.950** |
|  | (0.372) | (0.357) | (0.268) | (0.539) | (0.379) |
| Number of observations | 467 | 469 | 468 | 468 | 468 |

*Notes.*  Each panel of the table presents separate specification. Each column is a separate regression of the outcome specified in the header on the covariates listed in the rows. Eth=Ethiopia, Jord=Jordan, Pal=Palestine. See Table 1 for notes on covariates. Standard errors are clustered at the *subkebele* level in Ethiopia to account for sampling design, and at the individual level in Jordan and Palestine and presented in parentheses. In the Palestine-specific estimates, adolescents living in the West Bank and non-refugees are the reference group. The Palestine sample did not include any married male adolescents or youth. *** p<.01, ** p<.05, * p<.10
